# Metabolite Profile, Ruminal Methane Reduction, and Microbiome Modulating Potential of Seeds of *Pharbitis nil*

**DOI:** 10.3389/fmicb.2022.892605

**Published:** 2022-05-09

**Authors:** Rajaraman Bharanidharan, Krishnaraj Thirugnanasambantham, Ridha Ibidhi, Myunggi Baik, Tae Hoon Kim, Yookyung Lee, Kyoung Hoon Kim

**Affiliations:** ^1^Department of Agricultural Biotechnology, College of Agriculture and Life Sciences, Seoul National University, Seoul, South Korea; ^2^Department of Ecofriendly Livestock Science, Institutes of Green-Bio Science and Technology, Seoul National University, Pyeongchang, South Korea; ^3^Pondicherry Centre for Biological Science and Educational Trust, Villupuram, India; ^4^Department of Biotechnology, Saveetha School of Engineering, Saveetha Institute of Medical and Technical Sciences, Chennai, India; ^5^Department of International Agricultural Technology, Graduate School of International Agricultural Technology, Seoul National University, Pyeongchang, South Korea; ^6^National Institute of Animal Sciences, Rural Development Administration, Jeonju, South Korea

**Keywords:** methane, rumen, *in silico*, culture systems, PUFA, quercetin, *Entodinium caudatum*

## Abstract

We identified metabolites in the seeds of *Pharbitis nil* (PA) and evaluated their effects on rumen methanogenesis, fiber digestibility, and the rumen microbiome *in vitro* and *in sacco*. Four rumen-cannulated Holstein steers (mean body weight 507 ± 32 kg) were used as inoculum donor for *in vitro* trial and live continuous culture system for *in sacco* trial. PA was tested *in vitro* at doses ranging from 4.5 to 45.2% dry matter (DM) substrate. The *in sacco* trial was divided into three phases: a control phase of 10 days without nylon bags containing PA in the rumen, a treatment phase of 11 days in which nylon bags containing PA (180 g) were placed in the rumen, and a recovery phase of 10 days after removing the PA-containing bags from the rumen. Rumen headspace gas and rumen fluid samples were collected directly from the rumen. PA is enriched in polyunsaturated fatty acids dominated by linoleic acid (C18:2) and flavonoids such as chlorogenate, quercetin, quercetin-3-*O*-glucoside, and quinic acid derivatives. PA decreased (*p* < 0.001) methane (CH_4_) production linearly *in vitro* with a reduction of 24% at doses as low as 4.5% DM substrate. A quadratic increase (*p* = 0.078) in neutral detergent fiber digestibility was also noted, demonstrating that doses < 9% DM were optimal for simultaneously enhancing digestibility and CH_4_ reduction. *In sacco*, a 50% decrease (*p* = 0.087) in CH_4_ coupled with an increase in propionate suggested increased biohydrogenation in the treatment phase. A decrease (*p* < 0.005) in ruminal ammonia nitrogen (NH_3_-N) was also noted with PA in the rumen. Analysis of the rumen microbiome revealed a decrease (*p* < 0.001) in the Bacteroidetes-to-Firmicutes ratio, suggesting PA to have antiprotozoal potential. At the genus level, a 78% decrease in *Prevotella* spp. and a moderate increase in fibrolytic *Ruminococcus* spp. were noted in the treatment phase. *In silico* binding of PA metabolites to *cyclic GMP-dependent protein kinase* of *Entodinium caudatum* supported the antiprotozoal effect of PA. Overall, based on its high nutrient value and antiprotozoal activity, PA could probably replace the ionophores used for CH_4_ abatement in the livestock industry.

## Introduction

Ruminal CH_4_ production, in addition to its adverse effect on the environment, represents the loss of 3–10% of the gross energy intake of the animal (Appuhamy et al., 2016). Over the past 50 years, ruminant nutritionists have developed numerous strategies for enteric CH_4_ abatement (Beauchemin et al., 2020). A meta-analysis showed that dietary supplementation of ionophore antibiotics (e.g., monensin) decreases CH_4_ production by up to 15% and increases feed efficiency in beef cattle (Appuhamy et al., 2013). However, the use of ionophores has been banned in many countries, including South Korea, due to concern over their residues in livestock products (Maron et al., 2013; Lee et al., 2018). Similarly, consumer acceptance of 3-nitrooxypropanol, although a promising CH_4_ inhibitor, will influence the acceptance of inorganic synthetic compounds (Beauchemin et al., 2020). Therefore, studies have focused on plant-based organic strategies such as supplementing lipids and oil seeds rich in polyunsaturated fatty acids (PUFAs) or additives rich in flavonoids, tannins, and saponins, which reportedly reduce CH_4_ production (Hassan et al., 2020; Ku-Vera et al., 2020). Nevertheless, the adverse effects of PUFAs and other phyto-additives on nutrient digestibility, rumen fermentation, and animal performance have raised concerns over the feasibility of such strategies (Beauchemin et al., 2020). Natural seaweeds and seaweed bioactives can reduce CH_4_ production with fewer adverse effects on animal productivity (Abbott et al., 2020). However, concern over the safety of feeding bromoform-containing seaweeds to livestock, their associated toxicity to the environment (i.e., ozone depletion), and their net carbon footprint necessitates the discovery of new potential natural feed additives for CH_4_ abatement (Glasson et al., 2022).

Our previous *in vitro* screening study involving 152 plant extracts reported that ethanolic extracts from the seeds of *Pharbitis nil* (Pharbitis semen; PA) can decrease CH_4_ by up to 37% (Bharanidharan et al., 2021a). *Pharbitis nil* (Convolvulaceae) is an annual climbing herb widely distributed throughout Korea, Japan, and China, and PA is used as a purgative agent and in treating digestive disorders (Kim et al., 2020). Previous phytochemical investigations have reported the isolation of bioactive chemical constituents such as resin glycosides (Ono, 2017), monoterpenoids (Lee et al., 2017), diterpenoids (Ki et al., 2009; Woo et al., 2017), triterpenoid saponins (Jung et al., 2008), flavonoids, chlorogenic acid derivatives (Saito et al., 1994), and phenolic compounds (Kim et al., 2011) from different parts of *P. nil*. Similarly, we reported the enrichment of PUFAs in PA using gas chromatography-mass spectroscopy (GC-MS) without derivatization (Bharanidharan et al., 2021a). However, a complete secondary metabolite and fatty acid (FA) profile of PA is needed to understand their effects on methanogenesis and fermentation. Similarly, studies on the dose response effect of PA on CH_4_ production and fermentation characteristics are needed to optimize the additive dosage such that minimal adverse effects on rumen fermentation and nutrient digestibility are achieved. Furthermore, nutritional interventions with high dietary PUFAs should be evaluated, including their *in vivo* effect on the rumen microbiota based on their toxicity toward major cellulolytic bacteria (Beauchemin et al., 2020).

Artificial rumen replacements such as *in vitro* continuous or batch culture systems do not adequately simulate *in vivo* rumen gas and volatile fatty acid (VFA) production due to various factors such as lack of VFA absorption and feed passage *in vitro* (Krishnamoorthy et al., 2005; Amanzougarene and Fondevila, 2020). *In sacco* experimental approaches involving live continuous culture systems (LCCSs) have been used to assess the digestibility of feedstuffs (Krizsan et al., 2013). However, to the best our knowledge, only our previous work (Kim et al., 2016) has used this approach for the initial assessment of feed additives in terms of their effects on CH_4_ production and microbial population. Moreover, LCSS-based approaches have yet to be validated.

Here, we profiled PA metabolites using ultra-performance liquid chromatography high-resolution mass spectroscopy (UPLC-HRMS/MS) and GC-MS; evaluated the *in vitro* effect of ground PA on CH_4_ production, fermentation, and digestibility; and evaluated *in sacco* the effect of PA on CH_4_ production, fermentation, and rumen microbial dynamics using an LCCS.

## Materials and Methods

### Animals, Basal Diet, and Plant Material

Four cannulated Holstein steers (mean body weight 507 ± 32 kg), cared for in accordance with guidelines of the Animal Ethical Committee, Seoul National University, Republic of Korea (approval number SNU-210615-1), were used for the *in vitro* and *in sacco* trials. A diet comprising tall fescue hay and commercial concentrate pellets (33:67, w/w) was used as the basal diet of the donor steers and as substrate for *in vitro* incubations. The animals were fed 5 kg dry matter (DM) of the basal diet twice daily at 09:00 and 17:00 and had *ad libitum* access to fresh water. PA was purchased from a local market in Dongdaemun-gu, Seoul, Republic of Korea. The diet material and PA were dried in a forced-air oven at 65°C for 72 h to estimate the DM content, ground to pass through a 1-mm screen (Thomas Scientific Model 4, Swedesboro, NJ, United States), and stored at −20°C until *in vitro* incubations and chemical analyses. The ingredient and nutrient compositions of the basal diet/substrate are presented in Table 1, and its FA composition can be found in Supplementary Table 1.

**TABLE 1:** Ingredients and chemical composition of the basal diet.

Ingredient composition, g/kg DM	
**Concentrate**	
Broken corn	8.4
Wheat	112.2
Sodium bicarbonate	5.5
Rice bran	44.3
Salt	2.0
Molasses	17.9
Ammonium chloride	1.0
CMS	9.9
Corn flake	132.0
DDGS	70.0
Soybean Hull	11.9
Amaferm^1^	0.4
Corn Gluten Feed	132.0
Limestone	21.6
Palm kernel meal	96.8
Mineral-vitamin mixture^2^	1.3
**Roughage**	
Tall fescue hay	333.0

**Chemical Composition, g/kg DM**	

**Diet/Substrate**	
Organic matter (OM)	942.5
Crude protein (CP)	112.5
Ether extract (EE)	43.5
Neutral detergent fiber (aNDFom)^3^	400.5
Acid detergent fiber (ADFom)^4^	202.0
Gross energy (GE, MJ/kg)	18.5

*CMS, Condensed Molasses Soluble; DDGS, Dried Distiller’s Grains with Solubles.*

*^1^Amaferm: A fermentation extract of Aspergillus oryzae (Biozyme Enterprises Inc., MO, United States).*

*^2^Nutrients per kg of additive (Grobic-DC; Bayer HealthCare, Leverkusen, Germany): Vit. A, 2,650,000 IU; Vit. D3, 530,000 IU; Vit. E, 1,050 IU; Niacin, 10,000 mg; Mn, 4,400 mg; Zn, 4,400 mg; Fe, 13,200 mg; Cu, 2,200 mg; I, 440 mg; Co, 440 mg.*

*^3^Neutral detergent fiber assayed with a heat stable amylase and expressed exclusive of residual ash.*

*^4^Acid detergent fiber excluding residual ash.*

### Chemical Analyses

The feed and PA samples were dried in a forced-air oven at 65°C for 72 h to estimate DM content and then ground to pass through a 1-mm screen (Thomas Scientific Model 4). The organic matter content was determined after ashing at 600°C for 3 h using a Nabertherm LE 14/11/R7 Compact Muffle Furnace (Lilienthal, Germany) (Undersander et al., 1993). The ether extract (EE) content was determined using an ANKOM^XT15^ Extractor (Ankom Technology Corp., Fairport, NY, United States) following a filter bag procedure (AM-5-04; 2001) with petroleum ether as the solvent. Neutral detergent fiber (NDF) and acid detergent fiber contents were measured using a filter bag technique with an Ankom A2000 Fiber Analyzer (ANKOM Technology Corp.). The neutral detergent fiber content was analyzed using heat-stable amylase and expressed exclusive of residual ash (Van Soest et al., 1991). The analytical method for acid detergent fiber was based on Van Soest (1973), and the results are presented exclusive of residual ash. The nitrogen (N) content was determined using the Kjeldahl method (Kjeltec Auto Sampler System, 8400 Analyzer; Foss, Sweden) as described by (AOAC International, 2016). Crude protein (CP) was calculated as 6.25 × N. The gross energy content was determined using an automatic isoperibol calorimeter (6400EF, Parr Instrument Company, Moline, IL, United States). Concentrations of FAs in the feed and PA were analyzed using the direct methylation method of O’Fallon et al. (2007) and an Agilent 7890B GC system (Agilent Technologies, Santa Clara, CA, United States) with a flame ionization detector as described previously (Bharanidharan et al., 2021b). The FA content was expressed as mg/100 g DM.

### Metabolite Extraction and Sample Preparation for Ultra-Performance Liquid Chromatography High-Resolution Mass Spectroscopy

*Pharbitis nil* samples (10 g) were ground into a homogenous fine powder using a mortar and pestle in liquid nitrogen, followed by extraction with 2 L of methanol-water (80:20, v/v) with continuous stirring for 24 h at room temperature. The resultant extract was filtered using Whatman No. 2 filter paper, and the residue was re-extracted using 1 L of methanol-water (80:20, v/v) with continuous stirring for 12 h at room temperature. Both extracts were pooled and concentrated using a rotary vacuum evaporator (Heidolph Instruments, Schawabatch, Germany) followed by freeze drying (FDFC-12012, Operon, Seoul, South Korea) for 48 h. The dry extract (yield 3.2% w/w) was stored at −80°C until analysis. For UPLC-HRMS analysis, 20 mg of dry extract was dissolved in 2 mL of methanol-water (80:20, v/v) via sonication for 10 min (VCX130, SONICS Vibra-Cell™, Newtown, CT, United States), and the solution was passed through a 0.22-μm polyvinylidene fluoride syringe filter (Millex-GV, Millipore^®^, Darmstadt, Germany). The filtered samples were dried completely using a nitrogen evaporator (HyperVap HV-300, Labogene, Seoul, South Korea), and the dried extracts were reconstituted (5 mg/mL) in 80% HPLC-grade methanol (Sigma-Aldrich, St. Louis, MO, United States) in an autosample vial prior to analysis.

### Ultra-Performance Liquid Chromatography High-Resolution Mass Spectroscopy Analysis for Untargeted Metabolomics

UPLC separation was performed using an UltiMate™ 3000 (Thermo Scientific, Waltham, MA, United States) UPLC system with a CORTECS C18 (2.1 mm × 150 mm, 1.6 μm; Waters, Milford, MA, United States) UPLC column. The flow rate was 0.3 mL/min, and the solvent system consisted of 0.1% aqueous formic acid (A) and acetonitrile with 0.1% formic acid (B). Linear gradient elution was applied as follows: 1% B at 0–1.0 min, 30% B at 1.0–25.0 min, 30-100% B at 25–50 min, and 100-1% B at 50–60 min. The column temperature was held at 45°C, and the injection volume was 5 μL.

Electrospray ionization (ESI)-MS was carried out using a hybrid triple quadrupole time-of-flight (TripleTOF ^®^ 5600 + ; AB Sciex, Framingham MA, United States) mass spectrometer with MS1 and MS2 data recorded in both positive and negative ionization modes. The ion spray voltage in positive and negative mode was 5.5 and −4.5 kV, respectively. The desolvation gas (N_2_) temperature was set to 500°C. For MS acquisition, the nebulizer gas (N_2_), heating gas (N_2_), and curtain gas (N_2_) flow rates were 50, 50, and 25 psi, respectively. A declustering potential of ±60 V, collision energy of ±35 V, and collision energy spread of ±10 V were applied, in both positive and negative ionization modes. The MS analysis alternated between MS full scanning and information-dependent acquisition scanning.

Elements version 2.1.1 software (Proteome Software Inc., Portland, OR, United States) was used to process raw ion chromatograms. Raw data files were converted to mz5 format using ProteoWizard version pwiz_Reader_ABI: 3.0.9987. Raw profile data and converted data were imported into Elements software for peak identification, alignment, feature extraction, and area normalization, with separate analyses used for positive and negative ionization mode. Feature finding was conducted over a mass range of 30–1500 m/z as described previously (Lim, 2020).

### *In vitro* Effects of *Pharbitis nil* on CH_4_ Production and Digestibility

Approximately 300 mL of ruminal fluid was collected from each donor steer before the morning feeding and strained through four layers of muslin before getting pooled into a prewarmed flask flushed with O_2_-free CO_2_. The fluid was then diluted with O_2_-free buffer (McDougall, 1948; adjusted to pH 7.0) at a ratio of 1:2 (v/v) and placed in a water bath pre-heated to 39°C with continuous CO_2_ flushing. The *in vitro* trial was performed as described in Bharanidharan et al. (2021a) to test the effect of ground PA. Briefly, incubation was carried out with three replicates with each comprising 30 mL of rumen fluid mixture in 60-mL serum bottles containing 210 mg DM of substrate. The experimental setup comprised a blank (i.e., only rumen fluid mixture without substrate and ground PA), a control (i.e., rumen fluid mixture with substrate but without ground PA), a positive control (i.e., rumen fluid mixture with substrate and 30 ppm monensin; CAS No. 22373-78-0, Sigma-Aldrich), and dietary treatments (i.e., rumen fluid mixture with substrate and 4.5, 9.0, 13.6, 18.1, 22.6, or 45.2% DM ground PA). After 24 h of incubation, the total gas production was measured using water displacement apparatus (Fedorah and Hrudey, 1983). CH_4_ concentration in the headspace gas and VFAs in the medium were determined using the Agilent 7890B GC system (Agilent Technologies, Santa Clara, CA, USA) with a flame ionization detector using methods described by Bharanidharan et al. (2021a). The pH was measured using a pH meter (model AG 8603; Seven Easy pH, Mettler-Toledo, Schwerzenbach, Switzerland) and the NH_3_-N concentration was determined using a modified colorimetric method (Chaney and Marbach, 1962).

In parallel procedures, another fermentation run was conducted to determine the effects of PA on *in vitro* DM and NDF digestibility. Incubation was carried out with three replicates with each comprising 60 mL of rumen fluid mixture in 120-mL serum bottles containing 420 mg DM of substrate. The same experimental setup with the same dietary treatments as above was used for the trial. After 24 h of incubation, the incubation medium was transferred to 50-mL centrifuge tubes. Any particles attached to the walls of the serum bottles were washed off with distilled H_2_O and transferred to centrifuge tubes. The tubes were centrifuged at 3000 × *g* (ScanSpeed 1580R, Labogene, Seoul, South Korea) for 20 min, and the supernatants were discarded. The tubes containing the pellets were dried in a forced-air oven at 65°C for 48 h to determine the residual DM weights and NDF digestibility. The digestibility of DM and NDF was calculated based on the proportion of the initial weight incubated lost. The DM content of the blank was used for normalization. The incubation procedure was repeated three times in separate weeks to assess the repeatability.

### *In sacco* Effects of *Pharbitis nil* on CH_4_ Production and Rumen Microbial Dynamics

The same four cannulated Holstein steers used as rumen fluid donors were allocated to individual feeders equipped with steel stanchions, and the experiment was carried out as described previously (Kim et al., 2016). A total of twelve polyethylene/nylon bags (10 × 20 cm, pore size 300 ± 20 μm, FILTRA-BAG^®^, Mfr. No: EFT-1250A, Thomas Scientific) were filled with 60 g of unground PA each (three bags per steer). Each bag was sealed using a heat sealer and placed in a large retaining sac (20 × 30 cm, pore size 3 × 5 mm). A 3-m-long nylon cable was attached to one end of the retaining sac, and a cannula stopper was attached to the other end. The steers were fed the basal diet during a 14-day adaptation period before beginning the trial. The experiment was divided into three phases: a control phase of 10 days without sacs containing PA in the rumen, a treatment phase of 11 days in which three sacs containing PA were placed in three positions in the rumen (total 180 g per steer), and a recovery phase of 10 days after the sacs containing PA were removed from the rumen. The steers were locked in position by metal stanchions and allowed to stand until sampling was complete to minimize changes in ruminal volume and shape. Sampling for ruminal headspace gas was conducted on 11 occasions beginning 3 days before the initiation of the treatment phase (day 0). Gas samples from four steers were collected on days −3, −2, −1, 3, 5, 7, 9, 11, 14, 17, and 21 of the feeding period. On day 12, the steers began the recovery phase. Samples of rumen head space gas were taken at 1, 2, and 3 h after morning feeding by passing a 20-mL graduated syringe connected to a two-way stopcock (KOVAX, Seoul, South Korea) into the rumen through the cannula stopper. Six samples (three each during rumen contraction and relaxation) per sampling period were collected and immediately transferred to a vacuum tube (ref 364979, BD Vacutainer, Becton Dickinson, NJ, United States) for CH_4_ analysis as described previously (Bharanidharan et al., 2021a). Ruminal fluid samples were collected on days −1, 3, 11, and 21 at 3.5 h after morning feeding. After immediately measuring the pH using a pH meter (model AG 8603; Seven Easy pH, Mettler-Toledo, Schwerzenbach, Switzerland), ruminal fluid was transferred to a 50-mL centrifuge tube and centrifuged at 12,000 × *g* for 10 min (Supra-22K, Hanil Science Industrial, Gimpo, South Korea). The supernatant was transferred to a 15-mL centrifuge tube and stored at −20°C until analysis of NH_3_-N and VFA concentrations as described previously (Bharanidharan et al., 2018).

For microbial analysis, ruminal fluid samples collected on days −1, 11, and 21 were snap-frozen in liquid nitrogen and stored at −80°C until DNA extraction and next-generation sequencing using previously described methods and primers (Bharanidharan et al., 2018). Primers targeting the variable region V4 (515F/806R) were used because they cover both bacterial and archaeal populations, producing an amplicon size amenable for Illumina MiSeq sequencing (Caporaso et al., 2011; Kozich et al., 2013). The raw Illumina MiSeq reads generated were demultiplexed according to their barcodes, and the sequences were quality-filtered (≥Q20) based on the quality control process in Quantitative Insights into Microbial Ecology (QIIME) version 1.9.0 software with a filtered length of ≥ 200 bp as described previously (Bharanidharan et al., 2021c). The processed paired reads were concatenated into a single read, and each single read was screened for operational taxonomic unit (OTU) picking using the *de novo* clustering method CD-HIT-OTU embedded in QIIME 1.9.0 with reference to the National Center for Biotechnology Information (NCBI) database (NCBI_16S_20190602, 97% nucleotide identity).

### *In silico* Effects of *Pharbitis nil* Metabolites Against *Entodinium caudatum*

*Cyclic GMP (cGMP)-dependent protein kinase* (cGK), the central regulator of cGMP signaling in malaria parasites and a potential target for new antimalarial drugs (Baker et al., 2020), was used to evaluate the potential antiprotozoal effects of major PA metabolites. Because the rumen protozoa-specific protein sequence of cGK was not available in non-redundant databases, we used a manual method to determine the protein sequence. The partial mRNA sequence of cGK 9-1(XM_001017123) from *Tetrahymena thermophila* SB210, a widely used protozoal model, was retrieved from the NCBI database (accessed on 02 September 2021). The non-annotated genome assembly of *E. caudatum* MZG-1 (NBJL00000000.3) was downloaded from the NCBI database (accessed on 02 September 2021), and a local nucleotide database was created in BioEdit version 7.2.5 (Scripps Research, La Jolla, CA, United States) (Hall, 1999). A local nucleotide BLAST (BLASTn) search of the *T. thermophila* SB210 cGK nucleotide sequence was performed against the *E. caudatum* strain MZG-1 genome database. The result revealed nucleotide homology with *E. caudatum* strain MZG-1 (NBJL03004678.1). Furthermore, a protein homolog of the *E. caudatum* strain MZG-1 protein was searched for using the BLASTx algorithm^1^. The results revealed that the sequence of *E. caudatum* strain MZG-1 was 67% homologous (*e*-value = 0) to *Bacillus* cyclic nucleotide-binding domain-containing protein (MBP3841481) and its corresponding nucleotide (JAGAWU010000032) sequences. The corresponding aligned nucleotide sequence of *E. caudatum* strain MZG-1 was extracted and translated to an amino-acid sequence using the BLASTx algorithm. The amino-acid sequence was confirmed to encode cGK and was later used as the query for structure prediction.

The primary structural parameters of the *E. caudatum* strain MZG-1 specific cGK (query) protein were determined using ProtParam^2^. The physicochemical characters such as molecular weight, theoretical isoelectric point, amino-acid composition, extinct coefficient, estimated half-life, instability index, aliphatic index, and grand average of hydropathicity were computed. Secondary structure conformational parameters related to the presence of α-helices, β-turns, extended strands, and random coils were computed using the self-optimized prediction method with alignment (SOPMA) tool^3^. The similarity of the query protein with publicly available homologs was assessed by searching against non-redundant databases, including NCBI and Protein Data Bank (PDB) databases. The online protein structure prediction tool PS^2^^4^ was used to predict the three-dimensional structure of the protein. Superimposition of predicted models of the query onto the template was performed using SALIGN^5^ and visualized using the UCSF Chimera package (Pettersen et al., 2004). The models were analyzed based on the DOPE score, and their stereochemical quality was validated by generating a Ramachandran plot using PROCHECK^6^ on the SAVES server^7^.

For molecular docking studies, the chemical structures of the major metabolites (ligands) of PA (dicaffeoyl quinic acid, quercetin-3-*O*-glucoside, chlorogenate, palmitic acid, linoleic acid, and oleic acid) were retrieved from the PubChem compound database^8^. The retrieved ligand structures in .sdf format were converted to .pdb format using PyMOL version 1.7.4.5 software (DeLano Scientific LLC, San Carlos, CA, United States). Docking analysis was carried out using AutoDockTools version 1.5.4 and AutoDock version 4.2 software (Scripps Research Institute Molecular Graphics Laboratory, La Jolla, CA, United States) as described previously (Arokiyaraj et al., 2015). The docked complexes were visualized using Discovery Studio Visualizer version 4.5 (BIOVIA, San Diego, CA, United States).

### Statistical Analyses

*In vitro* data were analyzed using the PROC MIXED procedure of SAS version 9.4 (SAS Institute, Cary, NC, United States) with a Tukey-Kramer adjustment. The dose was considered a fixed effect and each incubation run a random effect. Linear, quadratic, and cubic components of the response to increasing doses of PA were evaluated using orthogonal polynomial contrasts. The CONTRAST option of the MIXED procedure uses the coefficient matrix generated in PROC IML for unequally spaced treatments. Pearson’s correlation coefficients were calculated to identify correlations between substrate FA concentration, CH_4_ production, fermentation characteristics, and digestibility using the PROC CORR function in SAS.

*In sacco* data were also analyzed using the PROC MIXED procedure of SAS. The statistical model for rumen microbial composition data included treatment type and period as fixed effects and each animal as a random effect. The headspace CH_4_ concentration, ruminal fermentation characteristics (pH, VFAs, and NH_3_), and microbial abundance were subjected to repeated-measures analysis of variance. The statistical model for fermentation parameters used the same model with the inclusion of day as a fixed effect. Within the period, each day was taken as a repeated measurement, and the treatment × day interaction was not evaluated. For the CH_4_ data, both day and time were included as fixed effects in the model and were considered repeated measurements. The treatment, day, and time effects as well as treatment × day and treatment × time interactions were evaluated. The Akaike information criterion was used to identify the covariance structure with the best fit, and the autoregressive and compound symmetry covariance structures were used to analyze the fermentation characteristics and CH_4_ data, respectively. Means were calculated using the LSMEANS function and were compared using the PDIFF option in SAS. Significant treatment effects were detected by pairwise comparisons employing Tukey’s test. Regarding differences according to treatment, *p* < 0.05 was taken to indicate significance and 0.05 < *p* < 0.1 was considered to indicate a trend toward significance. To identify bacterial lineages that drive the clustering of microbial communities in different phases, we performed principal component analysis (PCA) using the fviz_pca_biplot function in the FactoMineR package (Husson et al., 2020) in R version 4.0.3 software (The R Foundation for Statistical Computing, Vienna, Austria).

## Results

### *Pharbitis nil* Chemical Composition

The proximate and FA composition of PA are shown in Table 2. The CP, NDF, and EE contents of PA were 235.0, 421.0, and 129.4 g/kg DM, respectively. The analysis of the FA composition of PA revealed enrichment of saturated FAs (SFAs; 3.5 g/kg DM), monounsaturated fatty acids (MUFAs; 2.3 g/kg DM), and PUFAs (5.1 g/kg DM) dominated by linoleic acid (C18:2; 4.5 g/kg DM). The omega-6:omega-3 FA ratio was 10.2 with enrichment of omega-6 FAs (4.6 g/kg DM).

**TABLE 2:** Proximate and fatty acid composition of seeds of *Pharbitis nil*.

**Proximate composition (g/kg DM)**	
OM	948.0
CP	235.0
EE	129.4
aNDFom^1^	421.0
ADFom^2^	160.0
GE, MJ/kg	20.5
**Fatty acid composition (mg/100 g DM)**	
Caprylic acid (C8:0)	4.69
Capric acid (C10:0)	12.50
Myristic acid (C14:0)	23.81
Pentadecylic acid (C15:0)	4.03
Palmitic acid (C16:0)	2308.26
Palmitoleic acid (C16:1)	44.70
Margaric acid (C17:0)	10.44
Ginkgolic acid (C17:1)	0.61
Stearic acid (C18:0)	795.82
Elaidic acid (*trans* 9 C18:1)	30.79
Oleic acid (*cis* 9 C18:1)	2253.04
Trans-linoleic acid (C18:2n6t)	28.42
Linoleic acid (C18:2n6c)	4513.31
Arachidic acid (C20:0)	147.67
Gamma-Linolenic acid (C18:3n6)	60.80
Gondoic acid (C20:1n9)	7.07
Alpha linolenic acid (C18:3n3)	453.34
Heneicosylic acid (C21:0)	6.11
Eicosadienoic acid (C20:2n6)	8.29
Behenic acid (C22:0)	86.17
Tricosylic acid (C23:0)	2.33
Arachidonic acid (C20:4n6)	10.18
Lignoceric acid (C24:0)	51.39
SFA	3448.53
MUFA	2336.21
Omega-6	4620.99
Omega-3	453.34
Omega6:3	10.19
PUFA	5074.33
Trans fat	1534.24
Total fatty acids (mg/100 g DM)	10863.76

*^1^Acid detergent fiber expressed excluding residual ash.*

*^2^Neutral detergent fiber assayed with a heat stable amylase and expressed exclusive of residual ash.*

*OM, organic matter; CP, crude protein; EE, ether extract; GE, gross energy; SFA, saturated fatty acids; MUFA, mono unsaturated fatty acids; PUFA, poly unsaturated fatty acids.*

*SFA = C10:0 + C11:0 + C12:0 + C14:0 + C15:0 + C16:0 + C17:0 + C18:0 + C20:0 + C21:0 + C22:0 + C24:0.*

*MUFA = C14:1n5 + C16:1n7 + C17:1n7 + C18:1n7 + C18:1n9 + C20:1n9 + C22:1n9 + C24:1n9.*

*Omega-6 = C18:2n6 + C18:3n6 + C20:2n6 + C20:3n6 + C20:4n6 + C22:2n6 + C22:4n6. Omega-3 = C18:3n3 + C22:6n3.*

*PUFA = C18:2n6 + C18:2c9,t11 + C18:3n3 + C18:3n6 + C20:2n6 + C20:3n3 + C20:3n6 + C20:4n6 + C20:5n3 + C22:2n6 + C22:4n6 + C22:5n3 + C22:6n3.*

### *Pharbitis nil* Metabolite Profile

**FIGURE 1:**
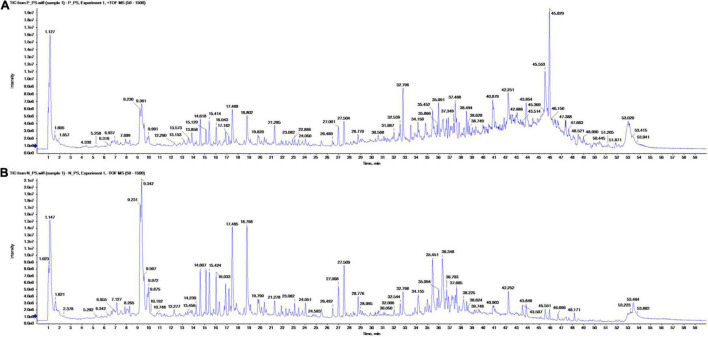
Total ion chromatograms of metabolites in the methanol extract of *Pharbitis nil* seeds in positive (A) and negative ionization (B) mode from ultra-performance liquid chromatography high-resolution mass spectroscopy analysis.

Altogether, 147 significant analytes were identified in the PA methanolic extract, 59 of which were identified in ESI positive ion mode (Figure 1A and Table 3), 54 in ESI negative ion mode (Figure 1B and Table 4), and 34 in both ESI positive and negative ion mode (Table 5). The identified analytes can be classified into saccharides and their derivatives, phenolic compounds and their derivatives, alkaloids, flavonoids, triterpenoids, coumarins, quinic acids, FAs, organosulfonic acids, and others. FAs and their derivatives, especially linoleic acid derivatives, were the main constituents in PA. Analytes such as caffeic acid, chlorogenate, quercetin, quercetin-3-*O*-glucoside, betaine, wilforlide A, 1,28-dicaffeoyloctacosanediol, hederagenin base + O-dHex-Hex-Hex, soyasaponin *V*, osmanthuside *H*, ginsenoside Ro, cinncassiol D2 glucoside, sarasinoside A1, and dioctyl sulfosuccinate were present in high proportions. Precursor organic acids including nicotinic acid, coumaric acids, dicaffeoylquinic acid, echinocystic acid, koetjapic acid, linoelaidic acid, and bovinic acid were identified. Finally, the phytosterols fucosterol, campesterol, and sitosterol were detected.

**TABLE 3:** Metabolites in the total methanolic extract of *P. nil* seeds identified using ultra-performance liquid chromatography high-resolution mass spectroscopy (UPLC-HRMS/MS) in positive ion mode.

Retention time (min)	Class	Analyte name	Formula	MW (g/mol)	log_10_ Ion Intensity
1.09	Antioxidant	Betaine	C_5_H_11_NO_2_	117.10	6.05
1.13	Saccharide	Sucrose	C_12_H_22_O_11_	342.10	5.84
1.55	Pyridine carboxylic acids	Nicotinic acid	C_6_H_5_NO_2_	123.00	5.75
2.00	Aromatic amino acid	Tyrosine	C_9_H_11_NO_3_	181.10	5.23
2.71	Ribonucleoside	Adenosine	C_10_H_13_N_5_O_4_	267.10	5.64
4.38	Aromatic amino acid	Phenylalanine	C_9_H_11_NO_2_	165.10	5.92
4.40	Aralkylamines	Phenylethanolamine	C_8_H_11_NO	137.10	5.66
9.22	Cinnamate ester and tannin	Chlorogenate	C_16_H_18_O_9_	354.10	5.84
9.57	Phenethyl alcohol glycosides	Darendoside A	C_19_H_28_O_11_	432.20	6.79
13.86	Hydroxycinnamic acids	*p*-coumaric acid	C_9_H_8_O_3_	164.00	5.44
15.13	Unknown	NCGC00380707-01_C26H42O11_(5xi,6alpha,7alpha,9xi,16xi)-16-(beta-D-Glucopyranosyloxy)-6,7,17-trihydroxykauran-19-oic acid	C_26_H_42_O_11_	530.30	6.09
15.41	Flavonoid	Quercetin	C_15_H_10_O_7_	302.00	5.47
16.89	Gamma-lactone	Marrubiin	C_20_H_28_O_4_	332.20	5.61
17.93	Hydroxycinnamic acids	*Cis*-caffeic acid	C_9_H_8_O_4_	180.00	5.30
21.29	Hydroxycinnamic acids	*p*-Coumaric acid	C_9_H_8_O_3_	164.00	5.42
22.90	Alkaloid	Ergocristine	C_35_H_39_N_5_O_5_	609.30	4.86
24.11	Unknown	Gabapentin related compound D	C_18_H_29_NO_3_	307.20	5.34
26.98	Triterpenoids	Jujubasaponin IV	C_48_H_78_O_18_	942.50	4.83
27.01	Triterpenoids	Echinocystic acid	C_30_H_48_O_4_	472.40	5.87
27.50	Phenolic glycosides	Wilforlide A	C_30_H_46_O_3_	454.30	5.63
28.76	Steroid acids	Koetjapic acid	C_30_H_46_O_4_	470.30	5.31
29.02	Fatty acyls	9-KODE	C_18_H_30_O_3_	294.20	5.17
29.02	LCFA	(Z)-5,8,11-trihydroxyoctadec-9-enoic acid	C_18_H_34_O_5_	330.20	5.68
32.52	Unknown	NCGC00385219-01_C17H20O4_3,6,9-Tris(methylene)-2-oxododecahydroazuleno[4,5-b]furan-8-yl acetate	C_17_H_20_O_4_	288.10	4.80
32.80	Coumaric acids and derivatives	1,28-Dicaffeoyloctacosanediol	C_46_H_70_O_8_	750.50	5.01
32.85	Fatty acyls	15-Ketoprostaglandin E1	C_20_H_32_O_5_	352.20	5.23
33.85	Amines	Phytosphingosine	C_18_H_39_NO_3_	317.30	5.39
33.87	MCFA	9(10)-Epoxy-12Z-octadecenoic acid	C_18_H_32_O_3_	296.20	5.62
34.32	LCFA	Vernolic acid	C_18_H_32_O_3_	296.20	6.35
34.54	LCFA	Linolenic acid	C_18_H_30_O_2_	278.20	5.99
34.38	Glycerophosphocholines	Lysophosphatidylcholine(18:3)	C_26_H_48_NO_7_P	517.30	4.66
35.42	Oxo fatty acid	12(13)Ep-9-KODE	C_18_H_30_O_4_	310.20	4.95
35.45	LCFA	Linoelaidic acid	C_18_H_32_O_2_	280.20	5.98
35.81	Monoglyceride	2-monopalmitin	C_19_H_38_O_4_	330.30	4.68
35.86	Amines	Desferrioxamine H	C_20_H_36_N_4_O_8_	460.30	6.44
35.98	Unknown	Asperhenamate_120258	C_32_H_30_N_2_O_4_	506.20	5.75
36.00	Glycerophosphocholines	1-Linoleoyl-lysophosphatidylcholine	C_26_H_50_NO_7_P	519.30	6.96
36.35	Triterpenoids	Ganoderic acid eta	C_30_H_44_O_8_	532.30	6.70
36.39	Glycerophosphocholines	1-palmitoyl-lysophosphatidylcholine	C_24_H_50_NO_7_P	495.30	5.77
36.85	Monoglyceride	Glyceryl linolenate	C_21_H_36_O_4_	352.30	5.83
37.48	Glycerophosphocholines	Oleoyl-lysophosphatidylcholine	C_26_H_52_NO_7_P	521.30	7.05
39.16	Fatty amide	Linoleoyl ethanolamide	C_20_H_37_NO_2_	323.30	5.68
39.33	Glycerophosphocholines	Stearoyl lysophosphatidylcholine	C_26_H_54_NO_7_P	523.40	6.35
40.86	Monoglyceride	1-Linoleoylglycerol	C_21_H_38_O_4_	354.30	6.44
40.93	Fatty amide	Oleoyl Ethanolamide	C_20_H_39_NO_2_	325.30	5.19
41.89	Glycerophosphocholines	1-eicosanoyl-sn-glycero-3-phosphocholine	C_28_H_58_NO_7_P	551.40	5.15
42.18	Unknown	Monoacyl glycerol	C_21_H_40_O_4_	356.30	5.47
42.23	Fatty acyls	Bovinic acid	C_18_H_32_O_2_	280.20	6.74
42.74	Chlorins	Pheophorbide A	C_35_H_36_N_4_O_5_	592.30	5.52
43.14	Unknown	NCGC00380823-01!2-(14-methylpentadecanoylamino)-3-phenylpropanoic acid	C_25_H_41_NO_3_	403.30	4.80
43.62	Fatty amide	*N*-Oleyl-Leucine	C_24_H_45_NO_3_	395.30	4.62
44.28	Ester	13S-Hydroxy-9Z,11E-octadecadienoic acid, methyl ester	C_19_H_34_O_3_	310.30	4.89
45.15	Stigmastanes	Fucosterol	C_29_H_48_O	412.40	5.70
45.77	Unknown	Digalactosyldiacylglycerols-36:6	C_51_H_84_O_15_	936.60	5.73
45.89	Ergostane steroids	Campesterol	C_28_H_48_O	400.40	5.37
45.94	Fatty amide	Erucamide	C_22_H_43_NO	337.30	7.64
46.73	Stigmastanes	Sitosterol	C_29_H_50_O	414.40	5.92
47.36	Triterpenoids	Friedelin	C_30_H_50_O	426.40	4.67
48.54	Diglycerides	Diacylglycerol(18:3n6/18:1n9)	C_39_H_68_O_5_	616.50	5.53

*MCFA, medium chain fatty acids; LCFA, long chain fatty acids.*

**TABLE 4:** Metabolites in the total methanolic extract of *P. nil* seeds identified using UPLC-HRMS/MS in negative ion mode.

Retention time (min)	Class	Analyte name	Formula	MW (g/mol)	log_10_ Ion Intensity
1.07	Saccharide	Sorbitol	C_6_H_14_O_6_	182.10	5.54
1.09	Saccharide	Gluconate	C_6_H_12_O_7_	196.10	6.58
1.12	Saccharide	Raffinose	C_18_H_32_O_16_	504.20	6.28
1.14	Saccharide	Sucrose	C_12_H_22_O_11_	342.10	6.24
2.16	Peptides	Glutathione disulfide	C_20_H_32_N_6_O_12_S_2_	612.20	4.61
6.96	Amino acids	Tryptophan	C_11_H_12_N_2_O_2_	204.10	5.39
9.09	Alkyl-phenylketones	3,4-Dihydroxyacetophenone	C_8_H_8_O_3_	152.00	4.78
9.12	Phenolic glycoside	Feruloyl Hexoside (isomer of 847)	C_16_H_20_O_9_	401.11	5.15
9.58	Coumaric acid esters	Osmanthuside H	C_19_H_28_O_11_	432.20	6.54
9.93	Cinnamate ester and tannin	Chlorogenate	C_16_H_18_O_9_	354.10	6.84
12.11	Aromatic amino acid tyrosine	Tyrosine	C_9_H_11_NO_3_	181.10	5.18
12.94	Phenyl propanoid	Coumarin + 1O	C_9_H_6_O_3_	162.00	4.67
15.12	Terpene glycosides	Cinncassiol D2 glucoside	C_26_H_42_O_11_	530.30	6.52
15.38	Lignan glycosides	Liriodendrin	C_34_H_46_O_18_	742.30	5.09
16.30	Flavanones	Silydianin	C_25_H_22_O_10_	482.10	6.12
17.19	Flavonoid glycosides	5,7-dihydroxy-6-[3,4,5-trihydroxy-6-(hydroxymethyl)oxan-2-yl]-8-[3,4,5-trihydroxy-6-(hydroxymethyl)oxan-2-yl]oxychromen-2-one	C_21_H_26_O_15_	518.10	5.22
18.16	Flavonoid glycosides	Isorhamnetin 3-galactoside	C_22_H_22_O_12_	478.10	5.38
18.79	Quinic acids	4,5-Dicaffeoylquinic acid	C_25_H_24_O_12_	516.10	6.01
19.78	Triterpene saponins	Soyasaponin V	C_48_H_78_O_19_	958.50	5.89
19.87	Phenolic glycoside	Lipedoside A	C_29_H_36_O_14_	608.20	6.04
20.92	Triterpene sapogenins	Soyasaponin Ba	C_48_H_78_O_19_	958.50	4.67
21.21	Quinic acids	4,5-Dicaffeoylquinic acid	C_25_H_24_O_12_	516.10	5.21
22.01	Triterpene saponins	Ginsenoside Ro	C_48_H_76_O_19_	956.50	5.12
23.43	Triterpene saponins	3-Glc-Gal-GlcUA-Soyasapogenol B	C_48_H_78_O_19_	958.50	5.78
23.77	Unknown	[(4R,5S,6R,6aS,7R,10aR,11bR)-5-acetyloxy-6-hydroxy-10a-methoxy-4,7,11b-trimethyl-9-oxo-1,2,3,4a,5,6,6a,7,11,11a-decahydronaphtho[2,1-f][1]benzofuran-4-yl]methyl acetate	C_25_H_36_O_8_	464.20	5.33
24.05	Triterpene glycosides	3-(Rha(1-2)Glu(1-2)Glu-28-Glu Hederagenin	C_54_H_88_O_23_	1104.60	5.78
25.47	Triterpene glycosides	3-Rha(1-2)Gal(1-2)GluA-Soyasaponenol B	C_48_H_78_O_18_	942.50	5.57
26.49	Fatty acid esters	26-(2-Glucosyl-6-acetylglucosyl]-1,3,11,22-tetrahydroxyergosta-5,24-dien-26-oate	C_42_H_66_O_17_	842.40	5.88
27.01	Triterpene saponins	Hederagenin base + O-dHex-Hex-Hex	C_48_H_78_O_18_	942.50	6.37
27.51	Unknown	NCGC00381017-01_C48H78O18_beta-D-Glucopyranose, 1-*O*-[(3beta,5xi,9xi)-3-[(6-deoxy-3-*O*-beta-D-glucopyranosyl-alpha-L-mannopyranosyl)oxy]-23-hydroxy-28-oxoolean-12-en-28-yl]	C_48_H_78_O_18_	942.50	6.49
28.34	Monoterpenoids	Soyasapogenol B base + O-HexA-HexA-Hex + Me + Acetyl	C_51_H_80_O_21_	1028.50	5.39
28.48	Monoterpenoids	Soyasapogenol B base + O-HexA-Hex	C_42_H_68_O_14_	796.50	4.89
28.83	Unknown	NCGC00169139-03_C42H66O15_1-*O*-[(3beta,5xi,9xi,18xi)-3-(beta-D-Glucopyranuronosyloxy)-23-hydroxy-28-oxoolean-12-en-28-yl]-beta-D-glucopyranose	C_42_H_66_O_15_	810.40	4.92
28.88	Triterpene saponins	Bayogenin base + O-Hex	C_36_H_58_O_10_	650.40	4.55
29.00	Fatty acyls	5-(oleoyloxy)octadecanoicacid	C_18_H_32_O_4_	312.20	6.15
30.31	Fatty acyls	9-HPODE_RT1	C_18_H_32_O_4_	312.20	5.96
30.58	Triterpene saponins	Elatoside K	C_53_H_84_O_23_	1088.50	5.03
32.01	Triterpene saponins	Camelliasaponin A1	C_58_H_92_O_25_	1188.60	5.40
32.24	Macrolide	Zearalenone	C_18_H_22_O_5_	318.10	5.09
32.81	Fatty acyls	13-HPODE	C_18_H_32_O_4_	312.20	6.12
34.21	Glycerophospholipids	LPI 18:3	C_27_H_47_O_12_P	594.30	5.75
35.19	Unknown	(E,2S,3R,4R,5S)-4-acetyloxy-2-amino-3,5,14-trihydroxyicos-6-enoic acid	C_22_H_41_NO_7_	431.30	6.03
35.48	Glycerophospholipids	LPI 18:2	C_27_H_49_O_12_P	596.30	6.89
35.99	Glycerophospholipids	LPS 21:1	C_27_H_52_NO_9_P	565.30	6.27
36.41	Glycerophospholipids	Lysophosphatidylcholine(16:0)	C_24_H_50_NO_7_P	495.30	5.01
36.70	LCFA	Linolenic acid	C_18_H_30_O_2_	278.20	6.31
36.64	Glycerophospholipids	LPI 18:1	C_27_H_51_O_12_P	598.30	5.64
36.80	Glycerophospholipids	LPG 18:2	C_24_H_45_O_9_P	508.30	5.19
37.05	Glycerophospholipids	LPC 18:1	C_26_H_52_NO_7_P	521.30	5.69
39.33	Glycerophospholipids	LPC 18:0	C_26_H_54_NO_7_P	523.40	5.62
39.64	Glycerophospholipids	LPI 18:0	C_27_H_53_O_12_P	600.30	5.86
42.25	Organosulfonic acid	Dioctyl sulfosuccinate	C_20_H_38_O_7_S	422.20	5.31
43.84	Organosulfonic acid	Dioctyl sulfosuccinate	C_20_H_38_O_7_S	422.20	4.77
47.66	LCFA	FAHFA 36:4	C_36_H_62_O_4_	558.50	5.21

*LCFA, long chain fatty acids.*

**TABLE 5:** Metabolites in the total methanolic extract of *P. nil* seeds identified using UPLC-HRMS/MS in both positive and negative ion modes.

Retention time (min)	Class	Analyte name	Formula	MW (g/mol)	log_10_ Ion Intensity	
					Positive	Negative
1.11	Aminoacids	Arginine	C_6_H_14_N_4_O_2_	174.10	6.64	4.88
1.62	Antioxidant	Citrate	C_6_H_8_O_7_	192.00	5.74	4.80
1.80	Alpha-amino acid	D-Glutamine	C_5_H_10_N_2_O_3_	146.10	5.99	5.12
1.93	Aminoacids	*N*-acetylglutamate	C_7_H_11_NO_5_	189.10	5.98	5.98
6.06	Vitamin	Vitamin B5	C_9_H_17_NO_5_	219.10	5.50	4.89
6.63	Purine nucleosides	*N6*-Succinyladenosine	C_14_H_17_N_5_O_8_	383.10	6.55	6.11
7.13	Cinnamate ester and a tannin	Chlorogenate	C_16_H_18_O_9_	354.10	6.26	6.52
9.37	Hydroxycinnamic acids	*Cis*-caffeic acid	C_9_H_8_O_4_	180.00	7.17	5.86
14.13	Antioxidant	Ferulate	C_10_H_10_O_4_	194.10	5.07	5.02
14.43	Unknown	NCGC00380707-01_C26H42O11_(5xi,6alpha,7alpha,9xi,16xi)-16-(beta-D-Glucopyranosyloxy)-6,7,17-trihydroxykauran-19-oic acid	C_26_H_42_O11	530.30	5.52	5.88
15.42	Antioxidant	Quercetin-3-*O*-glucoside	C_21_H_20_O_12_	464.10	6.18	6.46
16.03	Unknown	NCGC00380707-01_C26H42O11_(5xi,6alpha,7alpha,9xi,16xi)-16-(beta-D-Glucopyranosyloxy)-6,7,17-trihydroxykauran-19-oic acid	C_26_H_42_O_11_	530.30	6.03	6.50
16.05	Antioxidant	Quercetin 3-galactoside	C_21_H_20_O_12_	464.10	5.42	6.12
16.14	Antioxidant	Quercetin-4-*O*-glucoside	C_21_H_20_O_12_	464.10	5.42	6.15
17.49	Polyphenols	1,3-Dicaffeoylquinic acid	C_25_H_24_O_12_	516.10	7.14	7.17
17.50	Polyphenols	Dicaffeoyl quinolactone	C_25_H_22_O_11_	498.10	7.02	7.13
17.77	Flavonoid glycosides	Nepitrin	C_22_H_22_O_12_	478.10	5.08	5.40
18.82	Polyphenols	3,5-Dicaffeoylquinic acids	C_25_H_24_O_12_	516.10	6.93	7.11
18.99	Glycosyloxyisoflavone	Tectoridin	C_22_H_22_O_11_	462.10	5.73	5.59
32.78	Resinoside	Sarasinoside A1	C_62_H_100_N_2_O_26_	1288.70	5.74	5.95
33.89	MCFA	9(10)-Epoxy-12Z-octadecenoic acid	C_18_H_32_O_3_	296.20	5.35	6.02
34.15	LCFA	9,10-DiHOME	C_18_H_34_O_4_	314.20	6.10	6.44
35.67	Glycerophospholipids	1-Linoleoyl-lysophosphatidylserine	C_24_H_44_NO_9_P	521.30	5.12	5.22
35.73	Glycerophospholipids	1-Palmitoylglycerophosphoinositol	C_25_H_49_O_12_P	572.30	4.90	6.00
36.22	MCFA	9(10)-Epoxy-12Z-octadecenoic acid	C_18_H_32_O_3_	296.20	4.93	6.31
36.25	LCFA	Linolenic acid	C_18_H_30_O_2_	278.20	6.63	3.00
36.74	Glycerophoethanolamines	LysoPE(16:0/0:0)	C_21_H_44_NO_7_P	453.30	6.13	5.92
37.35	Glycerophoethanolamines	LysoPE(18:1(9Z)/0:0)	C_23_H_46_NO_7_P	479.30	6.26	5.96
37.38	Fatty acyls	9-KODE	C_18_H_30_O_3_	294.20	6.65	5.97
37.39	Fatty acyls	13-KODE	C_18_H_30_O_3_	294.20	6.65	5.97
37.60	Fatty acyls	Linoelaidic acid	C_18_H_32_O_2_	280.20	5.75	6.41
37.62	Fatty acyls	Bovinic acid	C_18_H_32_O_2_	280.20	6.14	6.41
38.23	Unknown	Dihydrocelastryl Diacetate	C_33_H_44_O_6_	536.30	3.08	5.50
39.15	Glycerophoethanolamines	LysoPE(18:0/0:0)	C_23_H_48_NO_7_P	481.30	5.30	5.25

*MCFA, medium chain fatty acids; LCFA, long chain fatty acids.*

### *In vitro* Effects of *Pharbitis nil* on CH_4_ Production, Fermentation, and Digestibility

Our results negate a dose–response relationship between PA and *in vitro* CH_4_ production, fermentation parameters, and digestibility (Table 6). Dietary SFA, MUFA, and PUFA concentrations increased with an increasing PA dose, whereas total gas production decreased linearly (*p* < 0.001). The production of CH_4_ (mmol/g DM) decreased by 23.7–69.1% compared with the control at doses of 4.5–45.2% DM. At doses ≤ 9% DM, enhancement of DM digestibility (DMD; *p* = 0.052) and NDF digestibility (NDFD; *p* = 0.073) by 9.6–15.2% was noted compared to the control, in which 31.1–50.5% decreases (*p* < 0.001) in CH_4_ production expressed as mmol/g digestible DM (CH_4_/dDM) or NDF (CH_4_/dNDF) were observed. At doses ≥ 13.6% DM, DMD and NDFD decreased but remained higher than in the control. Although DM and NDF degradation was unaffected at low doses of PA, the production of total VFAs and NH_3_-N decreased (*p* < 0.001). Conversely, the molar proportions of the VFAs propionate and butyrate increased linearly (*p* < 0.001) with an increasing dose. By contrast, the proportions of acetate, iso-butyrate, valerate, and iso-valerate decreased linearly (*p* < 0.001).

**TABLE 6:** Dose–response effect of *P. nil* seeds on *in vitro* methane (CH_4_) production, fermentation parameters, and digestibility (*n*_replicate_ = 4).

Item	Control	Monensin	*P. nil* seeds treatment (% substrate DM)						SEM	*p*-value	
			4.5	9.0	13.6	18.1	22.6	45.2		Linear	Quadratic
Total fatty acids, mg/g DM incubated	34.9	34.9	38.1	41.0	43.7	46.2	48.5	57.9			
*PUFA*	13.3	13.3	14.9	16.4	17.7	19.0	20.2	24.9			
*MUFA*	10.2	10.2	10.8	11.3	11.8	12.2	12.6	14.3			
*SFA*	11.4	11.4	12.4	13.3	14.2	15.0	15.7	18.6			
pH	6.3	6.4	6.4	6.4	6.4	6.4	6.4	6.4	0.01	0.030	0.175
Gas, mmol/g DM incubated	6.9	5.9	5.9	5.0	5.5	4.5	4.7	3.8	0.17	<0.0001	<0.0001
CH_4_, mmol/mol gas	162.9	142.9	145.8	127.6	121.0	105.2	101.1	88.3	2.57	<0.0001	<0.0001
CH_4_, mmol/g DM incubated	1.1	0.8	0.9	0.7	0.7	0.5	0.5	0.4	0.03	<0.0001	<0.0001
CH_4_, mmol/g NDF incubated	2.8	2.1	2.1	1.6	1.6	1.2	1.2	0.9	0.08	<0.0001	<0.0001
CH_4_, mmol/g DDM incubated	2.1	1.5	1.4	1.0	1.1	0.8	0.8	0.7	0.10	<0.0001	<0.0001
CH_4_, mmol/g DNDF incubated	6.4	4.7	4.5	3.2	3.3	2.5	2.6	2.0	0.34	<0.0001	<0.0001
Total VFA, mmol/g DM incubated	9.6	10.5	9.4	8.7	9.2	9.3	10.1	9.2	0.21	0.531	<0.0001
Acetate, %	47.8	46.0	47.9	46.1	44.5	39.6	34.6	33.2	1.05	<0.0001	0.338
Propionate, %	26.8	30.0	29.1	34.0	35.9	38.2	38.4	41.9	0.78	<0.0001	<0.0001
Isobutyrate, %	1.8	1.5	1.7	1.5	1.6	1.2	1.1	1.1	0.07	<0.0001	0.600
Butyrate, %	17.8	16.0	16.1	13.9	13.3	18.4	24.4	22.3	1.24	<0.0001	<0.0001
Isovalerate, %	4.7	5.6	4.1	3.6	3.9	2.2	1.2	1.2	0.32	<0.0001	0.304
Valerate, %	1.1	1.0	1.0	0.9	0.9	0.4	0.3	0.3	0.06	<0.0001	0.371
Acetate: propionate	1.8	1.6	1.7	1.4	1.2	1.1	0.9	0.8	0.05	<0.0001	<0.0001
NH_3_-N, mg/g DM incubated	41.9	38.6	37.4	27.7	28.7	29.2	34.3	27.1	0.64	<0.0001	<0.0001
DMD, mg/g DM incubated	550.7	572.0	603.8	634.6	610.7	603.4	587.8	536.2	25.65	0.472	0.052
NDFD, mg/g NDF incubated	437.0	457.4	480.1	504.0	485.4	479.2	466.8	426.4	28.41	0.627	0.073

*SFA, saturated fatty acids; MUFA, mono unsaturated fatty acids; PUFA, poly unsaturated fatty acids; NDF, neutral detergent fiber; DDM, digestible dry matter; DNDF, digestible NDF; DMD, dry matter digestibility; NDFD, NDF digestibility.*

Pearson’s correlation coefficients for the tested parameters are listed in Table 7. Strong negative associations of dietary FA concentrations with total gas production (*r* = −0.85; *p* < 0.005), total CH_4_ production (*r* = − 0.85; *p* < 0.005), and CH_4_ production per g dNDF (*r* = −0.76; *p* < 0.005) were noted *in vitro*. The dietary FA concentration was strongly associated with propionate production (*r* = 0.89; *p* < 0.005) and the NH_3_-N concentration (*r* = −0.71; *p* < 0.005). However, no association (*p* > 0.05) was noted between the dietary FA concentration and DMD or NDFD. The proportion of propionate was strongly negatively associated with total CH_4_ production (*r* = − 0.95; *p* < 0.005) and CH_4_ production per g dNDF (*r* = − 0.89; *p* < 0.005).

**TABLE 7:** Pearson correlation coefficients for associations between dietary fatty acid content, CH_4_ production, fermentation parameters, and digestibility.

	Gas, mmol/g DM	CH_4_, mmol/g DM	CH_4_, mmol/g DDM	CH_4_, mmol/g DNDF	Total VFA	Acetate	Propionate	DMD	NDFD	pH	NH_3_-N
Total fatty acids	−0.85**	−0.85**	−0.76**	−0.76**	–0.26	−0.88**	0.89**	–0.23	–0.19	0.13	−0.71**
SFA	−0.85**	−0.85**	−0.76**	−0.76**	–0.26	−0.88**	0.89**	–0.23	–0.19	0.13	−0.71**
MUFA	−0.85**	−0.85**	−0.76**	−0.76**	–0.26	−0.88**	0.89**	–0.23	–0.19	0.13	−0.71**
PUFA	−0.85**	−0.85**	−0.76**	−0.76**	–0.26	−0.88**	0.89**	–0.23	–0.19	0.13	−0.71**
NH_3_-N	0.81**	0.82**	0.82**	0.81**	0.57**	0.49*	−0.81**	–0.19	–0.14	–0.30	
pH	−0.49*	−0.46*	−0.56*	−0.52*	–0.10	–0.19	0.30	0.37†	0.26		
NDFD	–0.12	–0.09	–0.28	–0.34	–0.18	0.16	–0.04	0.86**			
DMD	–0.05	–0.08	–0.31	–0.30	–0.25	0.24	–0.04				
Propionate	−0.92**	−0.95**	−0.90**	−0.89**	–0.23	−0.87**					
Acetate	0.80**	0.83**	0.73**	0.73**	–0.10						
Total VFA	0.28	0.21	0.26	0.25							
CH_4_, mmol/g DNDF	0.95**	0.96**	0.99**								
CH_4_, mmol/g DDM	0.94**	0.97**									
CH_4_, mmol/g DM	0.97**										

*SFA, saturated fatty acids; MUFA, mono unsaturated fatty acids; PUFA, poly unsaturated fatty acids; DDM, digestible dry matter; DNDF, digestible NDF; DMD, dry matter digestibility; NDFD, NDF digestibility.*

^†^P < 0.1; *P < 0.05; **P < 0.005; ***P < 0.001.

### *In sacco* Effects of *Pharbitis nil* on the Rumen Headspace CH_4_ Concentration, Fermentation, and the Rumen Microbiome

**FIGURE 2:**
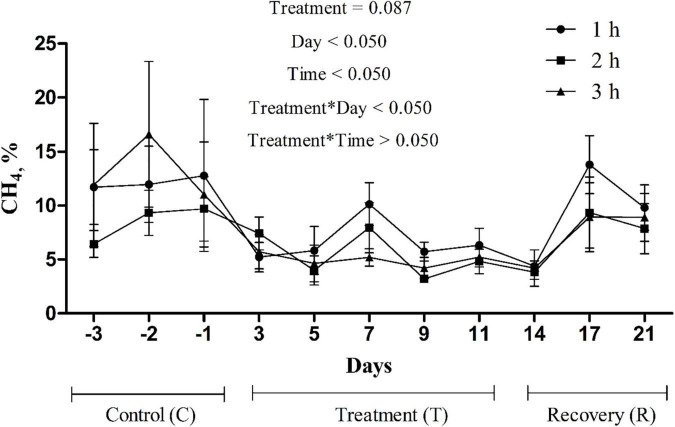
Changes in methane (CH_4_) concentration in rumen headspace gas due to the addition of *P. nil* seeds (*n* = 4 heads).

*In sacco*, the average CH_4_ concentration in the rumen headspace gas sample decreased (*p* = 0.087) from 11.3 to 5.5% in the treatment phase and then steadily increased to 8.9% in the recovery phase (Figure 2). An interaction between treatment and sampling day (*p* < 0.05) was noted for the CH_4_ concentration. No interaction (*p* > 0.05) between treatment and sampling time was noted. Ruminal fluid pH and NH_3_-N concentration decreased (*p* < 0.005) and remained relatively low at the end of the treatment phase, then increased in the recovery phase (Table 8). The total VFA concentration (*p* < 0.05) and the proportions of propionate (*p* = 0.089) and butyrate (*p* = 0.078) increased during the treatment phase and recovered toward the control level during the recovery phase. The acetate proportion exhibited the opposite pattern (*p* < 0.05).

**TABLE 8:** Effects of *P. nil* seeds on fermentation parameters *in sacco* in a live continuous culture system (*n* = 4 heads).

Item/Days	Control	Treatment		Recovery	SEM	*p*-value	
	Day -1	Day 3	Day 11	Day 21		Treatment	Day
Ruminal pH	6.8	6.6	6.1	6.6	0.06	0.051	0.001
Total VFA (mM)	94.0	105.5	126.1	106.3	2.50	0.013	0.003
Acetate, %	45.9	44.9	41.6	44.9	0.65	0.509	0.027
Propionate, %	27.9	29.6	30.3	28.9	0.96	0.180	0.400
Butyrate, %	17.2	17.2	20.8	17.5	0.73	0.913	0.012
Isobutyrate, %	1.7	1.5	1.0	1.6	0.09	0.435	0.003
Valerate, %	1.9	1.7	1.7	1.7	0.09	0.482	0.946
Isovalerate, %	5.5	5.2	4.6	5.3	0.39	0.658	0.232
Acetate: propionate	1.7	1.5	1.4	1.6	0.06	0.254	0.006
NH_3_-N, mg/dL	18.8	14.0	12.3	18.3	0.95	0.002	0.048

**FIGURE 3:**
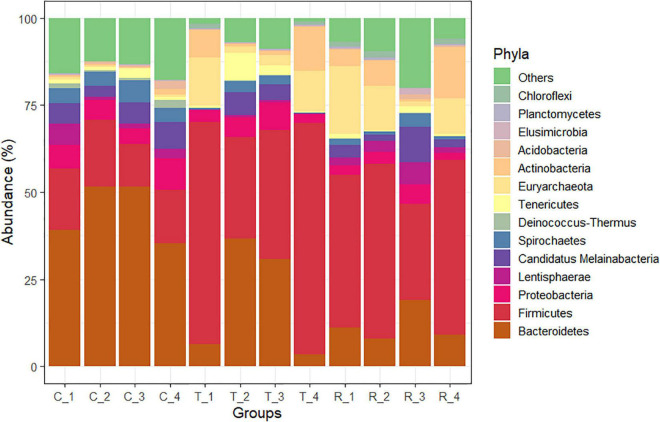
Phylum-level changes in the rumen microbiome of cannulated Holstein steers due to *P. nil* seeds. C, control; T, treatment; R, recovery.

**FIGURE 4:**
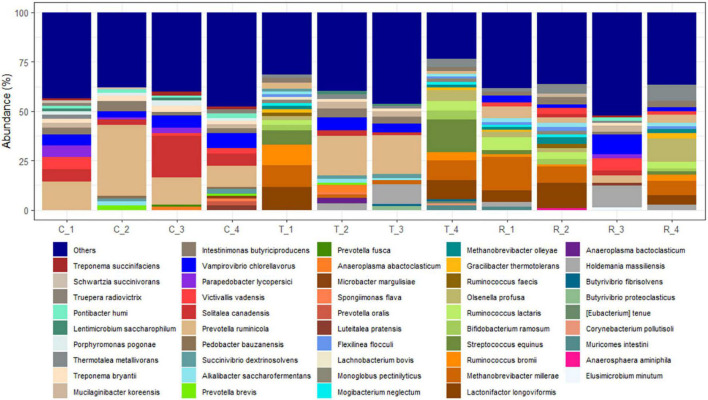
Species-level changes in the rumen microbiome of cannulated Holstein steers due to *P. nil* seeds. C, control; T, treatment; R, recovery.

**FIGURE 5:**
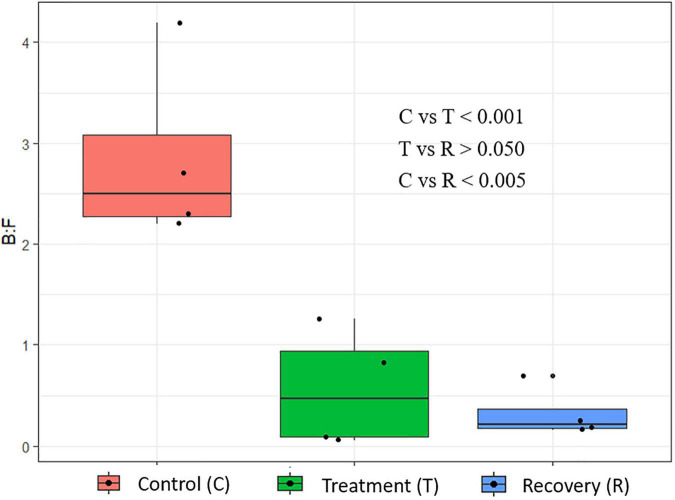
Changes in the Bacteroidetes:Firmicutes ratio in the rumen of cannulated Holstein steers due to *P. nil* seeds.

**FIGURE 6:**
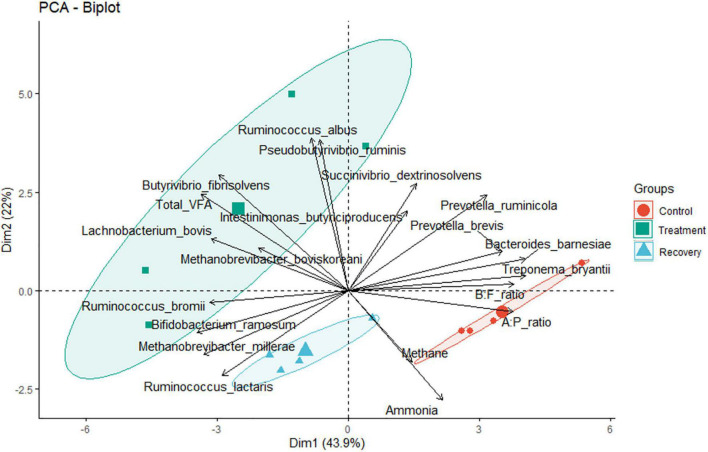
Principal component analysis results reflecting correlations between rumen bacterial/archaeal communities, CH_4_ yield, and fermentation parameters *in sacco*.

Analysis of the rumen microbial reads at a depth of 46,860 quality reads yielded an average of 886 OTUs (range 711–985 OTUs; Supplementary Figure 1). Diversity analysis of the rumen microbiota revealed that the total microbial species richness (alpha diversity) was unaffected by PA (Supplementary Figure 1). Analysis of the rumen microbial composition revealed marked shifts in relative abundance at the phylum and species levels in the post-treatment period (Figures 2, 3 and Supplementary Table 2). Decreased abundances of sequences assigned to the phyla Bacteroidetes (*p* < 0.05), Spirochaetes (*p* < 0.05), and Proteobacteria (*p* = 0.062) and an increase (*p* < 0.05) in the phylum Firmicutes were observed post-treatment (Figures 3, 4 and Supplementary Table 2). Consequently, a decrease (*p* < 0.005) in the Bacteroidetes:Firmicutes ratio was noted (Figure 5). Regarding beta diversity, PCA revealed that all rumen samples from steers at different phases clustered separately, with 43.9% of the variation explained (Figure 6). PA led to a 78% decrease (*p* < 0.05) in the abundance of OTUs assigned to the most abundant bacterial species, *Prevotella ruminicola* (18.1%), compared to the control phase (Supplementary Table 2). Other species in the families *Prevotellaceae* and *Paraprevotellaceae* exhibited decreases of similar magnitude. In terms of fibrolytic microorganisms, OTUs assigned to *Butyrivibrio fibrisolvens*, *Ruminococcus albus, Ruminococcus bromii*, and *Ruminococcus lactaris* increased (*p* < 0.05) several fold post-treatment (Supplementary Table 2). The abundance of *Streptococcus* sp. increased several-fold. Although the abundance of Phylum Euryarchaeota was unchanged, OTUs assigned to the genus *Methanobrevibacter* and *Methanosphaera* increased (*p* < 0.05) post-treatment (Supplementary Table 2).

### *In silico* Analysis of the Effects of Active Metabolites From *Pharbitis nil* on cGK

The results of a primary structural evaluation of the deduced cGK protein using ProtParam are shown in Supplementary Tables 3, 4. Evaluation of secondary structure conformational parameters using the SOPMA tool indicated that cGK is composed of 29.06% loops, 46.39% helices, 16.83% extended strands, and 7.72% β-turns (Supplementary Figure 2). Homology modeling of cGK performed based on the crystal structure of the A chain of *Plasmodium vivax* Sal-1 cGK (PDB ID: 4RZ7) as the template yielded sequence identity and *e*-values of 41.8 and 2e-94, respectively (Supplementary Figure 3A). The predicted model of cGK was superimposed on the cGK template (PDB ID: 4RZ7; Supplementary Figure 3B). PROCHECK analysis to assess the quality of the predicted model using a Psi/Phi Ramachandran plot indicated that 85.2% of the residues were in the most-favored regions, 10.1% were within additionally allowed regions, and 2.3% were in disallowed regions (Supplementary Table 5 and Supplementary Figure 3C). The G-factor for the modeled cGK was −0.27, revealing the predicted model to be of high quality (Supplementary Table 6). Therefore, model validation suggested that the model adequately represented the native protein.

**FIGURE 7:**
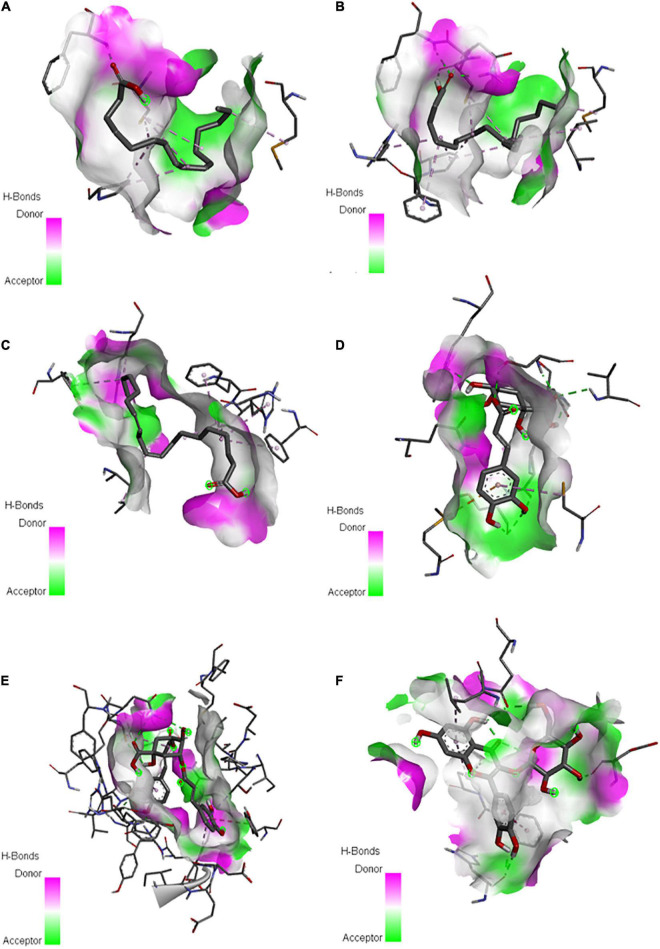
Putative binding sites of palmitic acid **(A)**, oleic acid **(B)**, linoleic acid **(C)**, chlorogenate **(D)**, dicaffeoyl quinic acid **(E)**, and quercetin-3-*O*-glucoside **(F)** from *P. nil* seeds with cyclic GMP-dependent protein kinase of the rumen protozoan *Entodinium caudatum.*

Analysis of docked protein-ligand complexes based on binding affinity revealed that the predicted protein model of *E. caudatum* cGK has the highest binding energies of −6.83, −6.75, and −5.92 kcal/mol with dicaffeoyl quinic acid, quercetin-3-*O*-glucoside, and chlorogenate, respectively (Supplementary Table 7). Long-chain FAs such as linoleic acid, oleic acid, and palmitic acid also exhibited fair binding to cGK with moderate energy values of −4.75, −4.52, and −4.14 kcal/mol, respectively (Supplementary Table 7). Hydrogen bond interactions between the ligands and the active residues of cGK were noted (Figures 7A–F and Supplementary Table 7).

## Discussion

Research on plant-based natural feed additives has been promoted due to restrictions on the non-therapeutic use of antibiotics in livestock production. Research into natural resources with high nutritional value and bioactive compounds can enhance productivity and mitigate the environmental footprint of livestock systems. Following our previous work (Bharanidharan et al., 2021a), we investigated for the first time the nutritive value and secondary metabolites of PA. We found high contents of CP, NDF, fat, and FAs in PA, suggesting that it has high nutritive value. The greater contents of FAs identified using GC-MS and FA derivatives identified using UPLC-HRMS/MS corroborate our prior finding (Bharanidharan et al., 2021a) that 60% of the compounds identified using GC-MS were derivatives of long-chain FAs. The fat content (129 g/kg DM) of PA is similar to that of grapeseed (Kapcsándi et al., 2021), whereas the FA profile (C16:0 [21%], C18:0 [21%], C18:2 [41%]) is comparable with that of sunflower seeds (Akkaya, 2018). Moreover, PA, as a source of omega-3 and -6 FAs (predominantly linoleic acid), can promote animal and human health (Haug et al., 2007). Plant secondary metabolites (PSMs) such as polyphenols reportedly benefit the health and productivity of ruminants (Olagaray and Bradford, 2019). The identification of flavonoids such as osmanthuside H, *p*-coumaric acid, quercetin, quercetin-3-*O*-glucoside, quercetin-4-*O*-glucoside, caffeic acid, ferulic acid, chlorogenic acid, and their derivatives in PA is consistent with previous studies (Saito et al., 1994; Kim et al., 2011). However, several metabolites (including betaine, wilforlide A, 1,28-dicaffeoyloctacosanediol, ginsenoside Ro, and other saturated and unsaturated FAs) are reported for the first time in this study. Notably, dioctyl sulfosuccinate, an anionic surfactant that is used as a laxative and stool softener, was identified in PA for the first time. PA is used in traditional medicine for its gastroprokinetic effect (Kim et al., 2020). In addition, the identified metabolites, including the FAs, have been shown to exhibit antimicrobial activity against bacteria (McGaw et al., 2002; Vasta et al., 2019) and protozoa (Matsumoto et al., 1991; Ayemele et al., 2020). Therefore, these compounds could be used to manipulate the rumen microbiome to mitigate CH_4_ emissions and optimize feed efficiency.

It is valuable to identify and assess the effects of metabolites present in plant materials on rumen fermentation characteristics and microbes than testing the raw plant material directly.

It is because the environmental factors such as soil conditions could greatly affect the concentration of the active metabolite in that plant. This in turn would influence the optimal dose determination and practical application of the plant material. However, it is also important to consider the synergic effects from other components which could be achieved when plant raw materials are used directly. Because the effects of crude PA extract was investigated at one dose in our previous study (Bharanidharan et al., 2021a), we used raw plant material at range of doses here. The decrease in CH_4_ production in response to PA *in vitro* confirms our previous observation (Bharanidharan et al., 2021a). At doses as low as 4.5% DM, PA decreased the production of CH_4_ (mmol/g DM incubated) by 24% *in vitro*, similar to 30 ppm monensin. The same effect was evident *in sacco*; the CH_4_ concentration in the rumen headspace gas decreased by 50% after 10 days. However, this could not be considered as a decrease in absolute CH_4_ production, since the total gas production was not quantified. This observed effect of PA on methanogenesis might have been related to the higher concentrations of FAs (3.8–5.8% DM) *in vitro*. The maximum decrease in CH_4_ production was 69% *in vitro*, and there were strong negative associations between the FA concentration and gas parameters, as reported previously (Patra, 2013). Patra (2013) suggested that for each percentage point increase in fat in the diet, CH_4_ emission decreased by 3.77% *in vivo* with a maximum reduction of 15.1% at 6% dietary DM, which is lower than our finding. It is possible that other PSMs in PA might have contributed to the effect (Ku-Vera et al., 2020). In addition, *in vitro* culture systems can overestimate such effects compared to *in vivo* systems (Machmüller, 2006).

The inhibitory effects of fats, FAs, and other PSMs on rumen methanogenesis are related to altered H_2_ thermodynamics in the rumen caused by a decrease in the populations of ciliate protozoa, their associated methanogens and other H_2_ producing bacteria (Newbold et al., 2015; Toprak, 2015). Although the protozoal population was not quantified in the current study, the decrease in methanogenesis in the current study could be attributed to the 90% decrease in total ciliate protozoan population as observed upon addition of ethanol extract of PA in our previous *in vitro* study (Bharanidharan et al., 2021a). Generally, rumen protozoa are associated with and engulf rumen bacteria and archaea (Park and Yu, 2018). Rumen protozoa degrade the proteins of engulfed microbial cells into oligopeptides and free amino acids, which are deaminated to produce free NH_3_-N, leading to inefficient dietary N utilization (Newbold et al., 2015). A recent study also reported that the presence or absence of different protozoal genera differentially modulate rumen prokaryotic community ecological structure (Solomon et al., 2021). Therefore, in this study, the shift in Bacteroidetes to Firmicutes ratio in the treatment phase could also be partially related to the defaunation effect of PA. However, other reasons including the specific binding of FAs in PA to the lipid bilayer membrane of Gram-negative bacteria that leads to cell death (McGaw et al., 2002) could also be attributed to the microbial shift. Huws et al. (2015) reported a 65% decrease in the *Prevotella* population upon adding C18:3-rich flax seed oil to the diet, supporting our hypothesis. Consistent with our results, Park et al. (2019) indicated a decrease in the Bacteroidetes:Firmicutes ratio as well as decreased *Prevotella* and increased *Streptococcus* abundances upon inhibition of *E. caudatum*. However, it could not be completely related to the results observed in the current study as different *Entodinium* spp. that exhibit different predatory behavior are represented in different proportions in the rumen (Kišidayová et al., 2021). Furthermore, the decrease in ruminal NH_3_-N in this study indicates reduced microbial protein turnover due to the inhibition of protozoa (Dai and Faciola, 2019). However, *Prevotella* species reportedly degrade dietary protein to produce NH_3_-N (Griswold et al., 1999); thus, the decrease in their relative abundance might have contributed to the reduced NH_3_-N. Although rumen protozoa population is associated with butyrate production, the increase in butyrate proportion upon adding PA *in vitro* may be partially related with the increase in relative abundance of butyrate producers such as *Butyrivibrio fibrisolvens* and *Pseudobutyrivibrio ruminis* in treatment phase of *in sacco* trial. The strong binding of PUFAs such as oleic acid, palmitic acid, and linoleic acid to cGK of *E. caudatum in silico* further provided insight into the antiprotozoal effect of PA, as these FAs are reportedly toxic to rumen protozoa (Dohme et al., 2008). This is the first study to predict the structure of *E. caudatum* cGK, which is involved in cell replication and other cell-cycle processes. Furthermore, in an evaluation of *Calotropis gigantea* extract rich in quercetin, quercetin-3-*O*-glucoside, and other hydrocinnamic acid derivatives for its effect on protozoa, Ayemele et al. (2021) reported a 50% decrease in the abundance of *E. caudatum* coupled with a decrease in NH_3_-N. This is consistent with the strong binding of quercetin-3-*O*-glucoside to *E. caudatum* cGK. Jin et al. (2021) reported the antimethanogenic potential of caffeic acid, in agreement with the large proportion of that flavonoid in PA in this study. Therefore, the major metabolites of PA may be responsible, at least in part, for inhibiting *E. caudatum*, thereby decreasing CH_4_ and NH_3_-N production.

Despite the symbiotic relationship between protozoa and methanogens (Levy and Jami, 2018), protozoal inhibition increased methanogen abundance, consistent with our previous study (Bharanidharan et al., 2021a). Similar responses to defaunation (Mosoni et al., 2011) and the addition of oils (Nur Atikah et al., 2018) have been reported. The proportion of methanogens that associates with protozoa is not large (Belanche et al., 2014), and other planktonic methanogens in the rumen might not be affected by PA. The decrease in CH_4_ caused by feed with increased lipid content is related to the increased production of propionic acid via biohydrogenation of unsaturated FAs (Hook et al., 2010). This is consistent with the strong associations between dietary PUFA content, propionate proportion, and CH_4_ production. This suggests that propionate synthesis was the major hydrogen sink despite the greater abundance of methanogens, which use H_2_ for CH_4_ production. Similarly, a monensin-mediated decrease in CH_4_ via increased biohydrogenation was caused by the inhibition of protozoa, whereas the effect on methanogens was minimal (Hook et al., 2010). In addition, the abundance of *B. fibrisolvens*, which is involved in the initial step of rumen biohydrogenation of PUFA to vaccenic acid (McKain et al., 2010), increased in the treatment phase. Moreover, decreased CH_4_ production due to linseed and coconut oil rich in PUFAs is reportedly not clearly linked to methanogenic gene abundance or changes in the archaeal community (Patra and Yu, 2013; Martin et al., 2016). It is also possible that the PA metabolites hindered the gene activity of methanogens for reducing H_2_ to CH_4_ in the rumen. However, after withdrawing PA (recovery phase) *in sacco*, the CH_4_ concentration in the headspace gas and other fermentation parameters started reverting to their initial values. This might have been due to the recovery of rumen microbes to their initial composition as a result of probable increase in rumen protozoal population in absence of PA. *In vitro* batch or continuous culture systems cannot be used to evaluate short- and long-term responses together with re-adaptation of microbes, and typically do not yield consistent results, unlike *in sacco* methods involving LCCSs. However, the outward and inward flow of gases through the cannula needs to be considered if this method is to be used to evaluate the effects of plant materials on methanogenesis and other fermentation parameters (Wang et al., 2019). In addition, lack of models for quantifying the total gas production has limited the LCCS approach to determine only the rumen headspace CH_4_ concentration rather than the absolute CH_4_ production.

Methane mitigation strategies involving plant additives are useful only if nutrient digestibility and other fermentation parameters are not concomitantly compromised. Dietary supplementation of lipids and the elimination of rumen protozoa are associated with decreased nutrient digestibility (Patra, 2013; Dai and Faciola, 2019). However, DMD and NDFD were increased by PA, likely because of the increased populations of fibrolytic bacteria such as *B. fibrisolvens*, *R. albus, R. bromii*, and *R. lactaris*. This is in agreement with the increase in *R. albus* and *R. flavefaciens* observed after defaunation (Mosoni et al., 2011; Park et al., 2019) and the addition of oil to a sheep diet (Adeyemi et al., 2016). Doreau et al. (2009) noted that dietary addition of oils did not negatively affect organic matter fermentation in the rumen, consistent with this work and a prior defaunation study (Park et al., 2019). It is also possibly a result of the protozoal groups inhibited; *Epidinium*, *Polyplastron*, and *Eudiplodinium* are cellulolytic, whereas *Entodinium* is weakly hemicellulolytic and so its contribution to fiber digestion is minimal (Takenaka et al., 2004). However, in this study, the magnitude of increase in digestibility declined at doses > 9% DM (>4.1% FAs), thereby suggesting that a dose of < 9% DM is optimal for decreasing CH_4_ production *in vitro* while enhancing digestibility. Moreover, meta-analyses (Hess et al., 2008; Knapp et al., 2014) have shown that dietary addition of up to 6% fats does not affect feed digestibility. Although an increase in digestibility was noted, the decrease in total VFA concentration and increase in pH suggest inhibition of rumen microbial fermentation *in vitro*, consistent with our previous study (Bharanidharan et al., 2021a). However, an inverse effect was observed *in sacco*—a decrease in ruminal pH and an increase in total VFAs. This variation in fermentation characteristics between culture systems further suggests the importance of using LCCSs, as an increase in the partial pressure of headspace gas inhibits fermentation in *in vitro* batch culture systems (Yang, 2017).

## Conclusion

Our results highlight the advantages of LCCSs for *in sacco* investigations over *in vitro* batch culture systems for studying fermentation characteristics. We determined the optimal dose of PA for decreasing CH_4_ production *in vitro* and the doses that modulate fermentation characteristics and digestibility. At doses < 9% DM, PA may be a potential additive for mitigating livestock CH_4_ emissions as it also increased the DMD and NDFD *in vitro*. PA contains bioactive compounds such as PUFAs and flavonoids that may reduce the rumen ciliate protozoan population, potentially decreasing intra-ruminal protein recycling and increasing biohydrogenation. However, re-adaptation of rumen microbes after the withdrawal of supplementation hampers commercial application of this strategy. Thus, the treatment duration and effects on individual protozoan genera should be addressed in future studies. Considering its high nutritive value, PA can be used in total mixed rations or as a substitute for grains in concentrate feed, making it a new source of functional feed for ruminants. However, *in vivo* studies are needed to evaluate other important nutritional traits, such as palatability, growth performance, nutrient digestibility, energy partitioning, and N utilization.

## Data Availability Statement

The datasets generated for this study can be found in online repositories. The name of the repository and accession number can be found below: NCBI, PRJNA789417 (https://www.ncbi.nlm.nih.gov/sra/PRJNA789417).

## Ethics Statement

The animal study was reviewed and approved by Institutional Animal Care and Use of Seoul National University (SNU-210615-1).

## Author Contributions

RB and KK designed and conceptualized the experiment. RB performed the management of steers, *in vitro* and *in sacco* trial, and sample collection. RB, KT, RI, and TK performed laboratory analyses. KT performed the *in silico* docking analysis. MB and YL supervised the experiment. RB organized the data, performed the microbial data processing, bioinformatics, statistical analyses and visualization. RB wrote the first draft of the manuscript including tables and figures, which was revised and edited by RB and KK. All authors read and approved the final manuscript.

## Conflict of Interest

The authors declare that the research was conducted in the absence of any commercial or financial relationships that could be construed as a potential conflict of interest.

## Publisher’s Note

All claims expressed in this article are solely those of the authors and do not necessarily represent those of their affiliated organizations, or those of the publisher, the editors and the reviewers. Any product that may be evaluated in this article, or claim that may be made by its manufacturer, is not guaranteed or endorsed by the publisher.

## Footnotes

1
http://blast.ncbi.nlm.nih.gov/Blast.cgi

2
https://web.expasy.org/protparam/

3
http://npsa-pbil.ibcp.fr/cgi-bin/npsa_automat.pl?page=/NPSA/npsa_sopma.html

4
http://ps2.life.nctu.edu.tw/

5
http://salilab.org/salign

6
http://www.ebi.ac.uk/thornton-srv/software/PROCHECK/

7
https://saves.mbi.ucla.edu/

8
https://pubchem.ncbi.nlm.nih.gov/
