# Supplementary material for: Metabolite Profile, Ruminal Methane Reduction, and Microbiome Modulating Potential of Seeds of Pharbitis nil
Source: Front Microbiol. 2022 May 9;13:892605. doi: 10.3389/fmicb.2022.892605 (PMC9125194; doi:10.3389/fmicb.2022.892605)
Supplement: Supplementary file 1 [file Data_Sheet_1.docx]

| **Fatty acid (mg/100 DM)** |  |
| --- | --- |
| Caprylic acid (C8 :0) | 1.81 |
| Capric acid (C10 :0) | 10.67 |
| Lauric acid (C12 :0) | 224.26 |
| Myristic acid (C14 :0) | 109.37 |
| Myristoleic acid (Cl4 :1) | 3.69 |
| Pentadecylic acid (C15 :0) | 3.29 |
| Palmitic acid (C16 :0) | 596.13 |
| Palmitoleic acid (C16 :1) | 32.93 |
| Margaric acid (C17 :0) | 7.43 |
| Stearic acid (C18 :0) | 168.34 |
| Elaidic acid (trans 9 C18 :1) | 0.35 |
| Oleic acid (cis 9 C18 :1) | 969.31 |
| Linoleic acid (C18 :2n6c) | 1215.11 |
| Arachidic acid (C20 :0) | 13.60 |
| Gondoic acid (C20 :1n9) | 14.89 |
| Alpha linolenic acid (C18 :3n3) | 102.17 |
| Heneicosylic acid (C21 :0) | 0.27 |
| Eicosadienoic acid (C20 :2n6) | 5.19 |
| Dihomo-gamma-linolenic acid (C20:3n6) | 0.78 |
| Tricosylic acid (C23 :0) | 1.67 |
| Arachidonic acid (C20 :4n6) | 3.20 |
| Lignoceric acid (C24:0) | 7.50 |
| Adrenic acid (C24 :1n9) | 1.40 |
| SFA | 1142.53 |
| MUFA | 1022.56 |
| Omega-6 | 1224.28 |
| Omega-3 | 102.17 |
| PUFA | 1326.45 |
| Trans fat | 0.35 |
| Total fatty acids (mg/100 g DM) | 3493.36 |

**Supplementary Table S1. Fatty acid composition of the basal diet and substrate used in this experiment.**

SFA, saturated fatty acids; MUFA, mono unsaturated fatty acids; PUFA, poly unsaturated fatty acids.

SFA = C10:0 + C11:0 + C12:0 + C14:0 + C15:0 + C16:0 + C17:0 + C18:0 + C20:0 + C21:0 + C22:0 + C24:0

MUFA = C14:1n5 + C16:1n7 + C17:1n7 + C18:1n7 + C18:1n9 + C20:1n9 + C22:1n9 + C24:1n9

Omega-6 = C18:2n6 + C18:3n6 + C20:2n6 + C20:3n6 + C20:4n6 + C22:2n6 + C22:4n6

Omega-3 = C18:3n3 + C22:6n3

PUFA = C18:2n6 + C18:2c9,t11 + C18:3n3 + C18:3n6 + C20:2n6 + C20:3n3 + C20:3n6 + C20:4n6 +  C20:5n3 + C22:2n6 + C22:4n6 + C22:5n3 + C22:6n3

**Supplementary Table S2. Relative abundance (%) of taxa in the rumen of cannulated Holstein steers incubated with seeds of *P. nil* representing > 0.1% of total sequences**

| **Phyla/Genera** | **Control (C)** | **Treatment (T)** | **Recovery (R)** | **SEM** | **P value** | | |
| --- | --- | --- | --- | --- | --- | --- | --- |
|  |  |  |  |  | **C vs. T** | **C vs. R** | **T vs. R** |
| ***Euryarchaeota*** | **0.56** | **7.54** | **10.97** | **8.70** | **0.439** | **0.339** | **0.822** |
| *Methanobrevibacter boviskoreani* | 0.03 | 0.32 | 0.39 | 0.15 | 0.082 | 0.040 | 0.665 |
| *Methanobrevibacter millerae* | 0.50 | 6.30 | 8.45 | 3.44 | 0.126 | 0.046 | 0.548 |
| *Methanobrevibacter olleyae* | 0.02 | 0.75 | 1.71 | 0.68 | 0.309 | 0.034 | 0.190 |
| *Methanosphaera stadtmanae* | 0.00 | 0.11 | 0.32 | 0.10 | 0.354 | 0.015 | 0.074 |
| ***Acidobacteria*** | **0.88** | **0.23** | **0.65** | **0.44** | **0.173** | **0.608** | **0.367** |
| *Luteitalea pratensis* | 0.88 | 0.23 | 0.65 | 0.44 | 0.173 | 0.608 | 0.367 |
| ***Actinobacteria*** | **0.63** | **5.61** | **6.75** | **3.40** | **0.177** | **0.105** | **0.744** |
| *Bifidobacterium ramosum* | 0.02 | 1.90 | 1.17 | 1.01 | 0.096 | 0.283 | 0.490 |
| *Corynebacterium pollutisoli* | 0.01 | 0.49 | 0.20 | 0.23 | 0.068 | 0.443 | 0.235 |
| *Atopobium parvulum* | 0.01 | 0.32 | 0.38 | 0.19 | 0.140 | 0.079 | 0.728 |
| *Olsenella profusa* | 0.32 | 2.07 | 4.17 | 2.29 | 0.466 | 0.128 | 0.383 |
| *Enterorhabdus muris* | 0.05 | 0.21 | 0.28 | 0.08 | 0.095 | 0.023 | 0.412 |
| ***Bacteroidetes*** | **44.39** | **19.30** | **11.78** | **7.96** | **0.012** | **0.003** | **0.369** |
| *Bacteroides barnesiae* | 0.16 | 0.05 | 0.05 | 0.04 | 0.022 | 0.022 | 1.000 |
| *Lentimicrobium saccharophilum* | 1.03 | 0.78 | 0.33 | 0.43 | 0.574 | 0.138 | 0.324 |
| *Muribaculum intestinale* | 0.36 | 0.09 | 0.16 | 0.13 | 0.063 | 0.137 | 0.635 |
| *Microbacter margulisiae* | 0.74 | 0.14 | 0.35 | 0.21 | 0.019 | 0.097 | 0.340 |
| *Porphyromonas pogonae* | 1.79 | 0.09 | 0.11 | 0.25 | <.0001 | <.0001 | 0.945 |
| *Marseilla massiliensis* | 0.11 | 0.23 | 0.07 | 0.10 | 0.249 | 0.673 | 0.129 |
| *Paraprevotella clara* | 0.25 | 0.17 | 0.08 | 0.08 | 0.342 | 0.052 | 0.250 |
| *Prevotella brevis* | 1.20 | 0.44 | 0.29 | 0.36 | 0.070 | 0.035 | 0.680 |
| *Prevotella fusca* | 0.65 | 0.08 | 0.24 | 0.22 | 0.033 | 0.105 | 0.498 |
| *Prevotella oralis* | 0.90 | 0.41 | 0.24 | 0.35 | 0.195 | 0.096 | 0.657 |
| *Prevotella ruminicola* | 18.71 | 11.14 | 4.08 | 6.32 | 0.262 | 0.046 | 0.292 |
| *Prevotellamassilia timonensis* | 0.16 | 0.05 | 0.05 | 0.05 | 0.041 | 0.058 | 0.833 |
| *Pontibacter humi* | 1.63 | 0.16 | 0.71 | 0.31 | 0.001 | 0.017 | 0.114 |
| *Rufibacter ruber* | 0.10 | 0.08 | 0.03 | 0.05 | 0.577 | 0.137 | 0.320 |
| *Spongiimonas flava* | 0.77 | 0.52 | 0.20 | 0.30 | 0.428 | 0.088 | 0.305 |
| *Mucilaginibacter koreensis* | 1.53 | 1.82 | 1.61 | 0.92 | 0.751 | 0.928 | 0.820 |
| *Parapedobacter deserti* | 0.13 | 0.09 | 0.18 | 0.08 | 0.633 | 0.507 | 0.267 |
| *Parapedobacter lycopersici* | 2.66 | 0.11 | 0.68 | 1.01 | 0.032 | 0.081 | 0.583 |
| *Pedobacter bauzanensis* | 0.93 | 0.13 | 0.08 | 0.13 | 0.000 | 0.000 | 0.674 |
| *Solitalea canadensis* | 9.09 | 1.14 | 1.22 | 3.42 | 0.045 | 0.047 | 0.982 |
| ***Candidatus Melainabacteria*** | **5.72** | **2.79** | **4.42** | **2.16** | **0.208** | **0.562** | **0.470** |
| *Vampirovibrio chlorellavorus* | 5.72 | 2.79 | 4.42 | 2.16 | 0.208 | 0.562 | 0.470 |
| ***Chloroflexi*** | **0.05** | **0.68** | **1.23** | **0.44** | **0.187** | **0.025** | **0.244** |
| *Flexilinea flocculi* | 0.05 | 0.66 | 1.22 | 0.43 | 0.194 | 0.025 | 0.231 |
| ***Deinococcus-Thermus*** | **1.14** | **0.12** | **0.11** | **0.31** | **0.009** | **0.009** | **0.962** |
| *Truepera radiovictrix* | 1.14 | 0.12 | 0.11 | 0.31 | 0.009 | 0.009 | 0.962 |
| ***Elusimicrobia*** | **0.21** | **0.14** | **0.61** | **0.34** | **0.835** | **0.271** | **0.199** |
| *Elusimicrobium minutum* | 0.14 | 0.07 | 0.46 | 0.26 | 0.811 | 0.250 | 0.174 |
| ***Firmicutes*** | **16.12** | **49.16** | **43.00** | **8.92** | **0.005** | **0.015** | **0.507** |
| *Salibacterium halotolerans* | 0.10 | 0.56 | 0.47 | 0.21 | 0.059 | 0.121 | 0.665 |
| *Novibacillus thermophilus* | 0.07 | 0.19 | 0.36 | 0.10 | 0.248 | 0.016 | 0.121 |
| *Planifilum composti* | 0.00 | 0.06 | 0.29 | 0.12 | 0.631 | 0.034 | 0.075 |
| *Streptococcus equinus* | 0.03 | 6.14 | 1.18 | 3.12 | 0.082 | 0.723 | 0.146 |
| *Colidextribacter massiliensis* | 0.06 | 0.18 | 0.10 | 0.09 | 0.221 | 0.698 | 0.384 |
| *Flintibacter butyricus* | 0.12 | 0.48 | 0.31 | 0.13 | 0.022 | 0.180 | 0.225 |
| *Intestinimonas butyriciproducens* | 3.12 | 3.10 | 2.63 | 0.90 | 0.989 | 0.604 | 0.613 |
| *Monoglobus pectinilyticus* | 0.09 | 0.92 | 0.65 | 0.43 | 0.086 | 0.226 | 0.545 |
| *Caloramator proteoclasticus* | 0.05 | 0.09 | 0.20 | 0.05 | 0.490 | 0.017 | 0.055 |
| *Clostridium cylindrosporum* | 0.26 | 0.04 | 0.05 | 0.08 | 0.023 | 0.027 | 0.926 |
| *Clostridium saudiense* | 0.02 | 0.29 | 0.16 | 0.14 | 0.079 | 0.335 | 0.361 |
| *Hungatella hathewayi* | 0.25 | 0.23 | 0.13 | 0.10 | 0.818 | 0.239 | 0.332 |
| *Lactonifactor longoviformis* | 0.12 | 5.33 | 5.82 | 3.23 | 0.142 | 0.112 | 0.883 |
| *Thermotalea metallivorans* | 0.89 | 2.46 | 3.89 | 1.46 | 0.310 | 0.070 | 0.355 |
| *[Eubacterium] sulci* | 0.01 | 0.09 | 0.15 | 0.07 | 0.251 | 0.063 | 0.396 |
| *Aminipila butyrica* | 0.17 | 0.07 | 0.11 | 0.03 | 0.015 | 0.115 | 0.248 |
| *Ihubacter massiliensis* | 0.07 | 0.43 | 0.64 | 0.21 | 0.119 | 0.025 | 0.359 |
| *Mogibacterium neglectum* | 0.03 | 0.80 | 0.88 | 0.41 | 0.095 | 0.070 | 0.856 |
| *Vallitalea pronyensis* | 0.37 | 0.34 | 0.17 | 0.10 | 0.780 | 0.069 | 0.109 |
| *Alkalibacter saccharofermentans* | 0.95 | 1.40 | 1.73 | 0.45 | 0.348 | 0.118 | 0.481 |
| *Eubacterium coprostanoligenes* | 0.15 | 0.18 | 0.46 | 0.18 | 0.902 | 0.117 | 0.142 |
| *Eubacterium ruminantium* | 0.16 | 0.19 | 0.03 | 0.07 | 0.626 | 0.133 | 0.060 |
| *Eubacterium ventriosum* | 0.05 | 0.29 | 0.29 | 0.18 | 0.213 | 0.204 | 0.978 |
| *Gracilibacter thermotolerans* | 0.37 | 1.08 | 1.30 | 0.52 | 0.208 | 0.108 | 0.677 |
| *Anaerobacterium chartisolvens* | 0.12 | 0.15 | 0.08 | 0.04 | 0.464 | 0.365 | 0.120 |
| *Saccharofermentans acetigenes* | 0.62 | 0.61 | 0.43 | 0.15 | 0.947 | 0.217 | 0.240 |
| *Thermoclostridium caenicola* | 0.20 | 0.01 | 0.07 | 0.05 | 0.003 | 0.027 | 0.218 |
| *Abyssivirga alkaniphila* | 0.16 | 0.21 | 0.20 | 0.02 | 0.071 | 0.185 | 0.554 |
| *Acetatifactor muris* | 0.12 | 0.30 | 0.32 | 0.12 | 0.181 | 0.146 | 0.891 |
| *Anaerobium acetethylicum* | 0.26 | 0.15 | 0.32 | 0.08 | 0.200 | 0.452 | 0.058 |
| *Anaerobutyricum hallii* | 0.02 | 0.27 | 0.51 | 0.20 | 0.234 | 0.033 | 0.243 |
| *Blautia hydrogenotrophica* | 0.00 | 0.08 | 0.23 | 0.12 | 0.507 | 0.078 | 0.227 |
| *Blautia marasmi* | 0.04 | 0.08 | 0.12 | 0.03 | 0.229 | 0.033 | 0.254 |
| *Butyrivibrio fibrisolvens* | 0.07 | 0.99 | 0.18 | 0.04 | <.0001 | 0.037 | <.0001 |
| *Butyrivibrio proteoclasticus* | 0.67 | 0.83 | 0.53 | 0.31 | 0.607 | 0.672 | 0.357 |
| *Hespellia porcina* | 0.10 | 0.14 | 0.13 | 0.03 | 0.150 | 0.276 | 0.688 |
| *Kineothrix alysoides* | 0.06 | 0.20 | 0.19 | 0.06 | 0.059 | 0.072 | 0.907 |
| *Lachnobacterium bovis* | 0.00 | 0.80 | 0.03 | 0.21 | 0.004 | 0.898 | 0.005 |
| *[Clostridium] lavalense* | 0.12 | 0.03 | 0.12 | 0.04 | 0.088 | 0.870 | 0.067 |
| *[Clostridium] scindens* | 0.11 | 0.29 | 0.23 | 0.09 | 0.073 | 0.198 | 0.539 |
| *Muricomes intestini* | 0.01 | 0.74 | 0.62 | 0.50 | 0.175 | 0.250 | 0.814 |
| *Pseudobutyrivibrio ruminis* | 0.11 | 0.40 | 0.15 | 0.16 | 0.096 | 0.770 | 0.154 |
| *Syntrophococcus sucromutans* | 0.02 | 0.30 | 0.39 | 0.16 | 0.119 | 0.045 | 0.563 |
| *Oscillibacter ruminantium* | 0.15 | 0.15 | 0.16 | 0.11 | 0.964 | 0.891 | 0.855 |
| *[Eubacterium] tenue* | 0.06 | 0.52 | 0.32 | 0.23 | 0.084 | 0.310 | 0.411 |
| *Romboutsia timonensis* | 0.04 | 0.41 | 0.20 | 0.19 | 0.087 | 0.408 | 0.320 |
| *Papillibacter cinnamivorans* | 0.23 | 0.04 | 0.09 | 0.07 | 0.021 | 0.064 | 0.514 |
| *Ruminococcus albus* | 0.04 | 0.22 | 0.09 | 0.07 | 0.046 | 0.582 | 0.115 |
| *Ruminococcus bromii* | 0.08 | 3.87 | 1.33 | 1.97 | 0.086 | 0.539 | 0.231 |
| *Ruminococcus faecis* | 0.00 | 0.69 | 0.85 | 0.50 | 0.196 | 0.122 | 0.762 |
| *Ruminococcus gauvreauii* | 0.06 | 0.17 | 0.09 | 0.06 | 0.085 | 0.702 | 0.158 |
| *Ruminococcus lactaris* | 0.09 | 1.83 | 3.36 | 1.43 | 0.255 | 0.048 | 0.314 |
| *Sporobacter termitidis* | 0.12 | 0.12 | 0.18 | 0.03 | 0.885 | 0.107 | 0.084 |
| *Breznakia pachnodae* | 0.27 | 0.39 | 0.22 | 0.17 | 0.511 | 0.784 | 0.358 |
| *Holdemania filiformis* | 0.10 | 0.39 | 0.31 | 0.07 | 0.003 | 0.016 | 0.310 |
| *Holdemania massiliensis* | 0.40 | 3.55 | 4.01 | 2.68 | 0.271 | 0.212 | 0.868 |
| *Turicibacter sanguinis* | 0.03 | 0.37 | 0.17 | 0.15 | 0.054 | 0.372 | 0.232 |
| *Succiniclasticum ruminis* | 0.37 | 0.34 | 0.23 | 0.11 | 0.783 | 0.253 | 0.372 |
| *Schwartzia succinivorans* | 0.90 | 0.25 | 0.51 | 0.21 | 0.012 | 0.087 | 0.254 |
| *Sedimentibacter acidaminivorans* | 0.14 | 0.10 | 0.12 | 0.05 | 0.468 | 0.696 | 0.732 |
| *Anaerosphaera aminiphila* | 0.31 | 0.58 | 0.52 | 0.20 | 0.198 | 0.323 | 0.739 |
| ***Lentisphaerae*** | **2.80** | **0.36** | **3.34** | **1.32** | **0.097** | **0.692** | **0.050** |
| *Victivallis vadensis* | 2.73 | 0.34 | 3.31 | 1.31 | 0.099 | 0.667 | 0.048 |
| ***Proteobacteria*** | **6.49** | **4.89** | **3.45** | **1.43** | **0.291** | **0.062** | **0.338** |
| *Kiloniella antarctica* | 0.05 | 0.16 | 0.22 | 0.10 | 0.309 | 0.117 | 0.530 |
| *Kiloniella laminariae* | 0.08 | 0.26 | 0.16 | 0.14 | 0.238 | 0.583 | 0.505 |
| *Ochrobactrum rhizosphaerae* | 0.22 | 0.02 | 0.06 | 0.04 | 0.001 | 0.005 | 0.423 |
| *Rhizobium skierniewicense* | 0.06 | 0.13 | 0.10 | 0.02 | 0.010 | 0.054 | 0.321 |
| *Aestuariispira insulae* | 0.13 | 0.29 | 0.19 | 0.17 | 0.369 | 0.724 | 0.575 |
| *Aliidongia dinghuensis* | 0.09 | 0.11 | 0.22 | 0.07 | 0.766 | 0.082 | 0.133 |
| *Sphingomonas olei* | 0.19 | 0.11 | 0.13 | 0.05 | 0.163 | 0.251 | 0.775 |
| *Pigmentiphaga aceris* | 0.10 | 0.07 | 0.07 | 0.03 | 0.463 | 0.320 | 0.781 |
| *Desulfobulbus elongatus* | 0.29 | 0.01 | 0.02 | 0.08 | 0.006 | 0.008 | 0.826 |
| *Ruminobacter amylophilus* | 0.20 | 0.13 | 0.03 | 0.08 | 0.407 | 0.054 | 0.209 |
| *Succinivibrio dextrinosolvens* | 1.34 | 1.29 | 0.41 | 0.44 | 0.900 | 0.065 | 0.080 |
| *Erwinia rhapontici* | 0.47 | 0.16 | 0.17 | 0.11 | 0.022 | 0.025 | 0.931 |
| *Chelonobacter oris* | 0.32 | 0.08 | 0.01 | 0.08 | 0.019 | 0.006 | 0.459 |
| *Pseudomonas reidholzensis* | 0.69 | 0.22 | 0.17 | 0.11 | 0.003 | 0.001 | 0.685 |
| *Luteimonas terrae* | 0.56 | 0.25 | 0.29 | 0.12 | 0.031 | 0.057 | 0.722 |
| *Stenotrophomonas tumulicola* | 0.22 | 0.25 | 0.24 | 0.10 | 0.784 | 0.842 | 0.940 |
| ***Spirochaetes*** | **4.72** | **1.60** | **1.84** | **0.98** | **0.011** | **0.017** | **0.809** |
| *Sphaerochaeta globosa* | 0.16 | 0.08 | 0.14 | 0.09 | 0.400 | 0.825 | 0.529 |
| *Treponema bryantii* | 2.45 | 0.64 | 0.62 | 0.39 | 0.001 | 0.001 | 0.961 |
| *Treponema pectinovorum* | 0.05 | 0.13 | 0.25 | 0.11 | 0.552 | 0.120 | 0.299 |
| *Treponema porcinum* | 0.17 | 0.08 | 0.09 | 0.06 | 0.173 | 0.220 | 0.873 |
| *Treponema ruminis* | 0.18 | 0.10 | 0.07 | 0.08 | 0.387 | 0.192 | 0.628 |
| *Treponema stenostreptum* | 0.09 | 0.15 | 0.15 | 0.09 | 0.568 | 0.533 | 0.956 |
| *Treponema succinifaciens* | 1.35 | 0.31 | 0.40 | 0.27 | 0.004 | 0.007 | 0.747 |
| ***Tenericutes*** | **1.38** | **2.89** | **1.02** | **1.46** | **0.326** | **0.811** | **0.231** |
| *Anaeroplasma abactoclasticum* | 0.65 | 1.19 | 0.50 | 0.76 | 0.499 | 0.841 | 0.386 |
| *Anaeroplasma bactoclasticum* | 0.22 | 0.94 | 0.20 | 0.53 | 0.212 | 0.964 | 0.198 |
| *Spiroplasma apis* | 0.15 | 0.05 | 0.05 | 0.03 | 0.005 | 0.007 | 0.803 |
| **Others** | **14.58** | **4.40** | **10.31** | **3.21** | **0.011** | **0.216** | **0.100** |

**Supplementary Table S3.** **Primary structural properties of *Entodinium caudatum* cGMP-dependent protein kinase**

| Parameters | | Theoretical Prediction |
| --- | --- | --- |
| 1. | Molecular weight (kDa) | 117.36 |
| 2. | Isoelectric point | 9.04 |
| 3. | Extinction coefficient (M^-1^ cm^-1^ at 280nm) | 79495 |
| 4. | Estimated half-life (h):  Mammalian reticulocytes (in-vitro)  Yeast (in-vivo)  *Escherichia coli* (in-vivo) | 30  >20  >10 |
| 5. | Instability index | 34.36 |
| 6. | Aliphatic index | 88.78 |
| 7. | Grand average of hydropathicity (GRAVY) | -0.460 |

**Supplementary Table S4.** **Amino acid composition of *Entodinium caudatum* cGMP-dependent protein kinase.**

| Amino acid | composition | Percentage % |
| --- | --- | --- |
| Ala (A) | 28 | 2.8 % |
| Arg (R) | 27 | 2.7% |
| Asn (N) | 90 | 9.0% |
| Asp (D) | 55 | 5.5% |
| Cys (C) | 14 | 1.4% |
| Gln (Q) | 32 | 3.2% |
| Glu (E) | 79 | 7.9% |
| Gly (G) | 33 | 3.3% |
| His (H) | 14 | 1.4% |
| Ile (I) | 105 | 10.5% |
| Leu (L) | 86 | 8.6% |
| Lys (K) | 132 | 13.2% |
| Met (M) | 26 | 2.6% |
| Phe (F) | 72 | 7.2% |
| Pro (P) | 25 | 2.5% |
| Ser (S) | 57 | 5.7% |
| Thr (T) | 42 | 4.2% |
| Trp (W) | 4 | 0.4% |
| Tyr (Y) | 38 | 3.8% |
| Val (V) | 39 | 3.9% |

**Supplementary Table S5.** **Ramachandran plot statistics of predicted three dimensional model of *Entodinium caudatum* cGMP-dependent protein kinase.**

| S.No | Ramachandran Plot statistics | No. of residues (percentage) |
| --- | --- | --- |
| 1. | Most favoured regions [A,B,L] | 799 (85.2 %) |
| 2. | Additionally allowed regions [a,b,l,p] | 95 (10.1 %) |
| 3. | Generously allowed regions [_˜_a, _˜_b, _˜_l, _˜_p] | 22 (2.3%) |
| 4. | Disallowed regions [XX] | 22 (2.3 %) |
| 5. | Total non-glycine and non-proline residues | 938 (100 %) |
| 6. | End-residues (excl. Gly and proline) | 2 |
| 7. | Glycine residues | 33 |
| 8. | Proline residues | 25 |
| 9. | Total number of residues | 998 |

**Supplementary Table S6.** **G-factor parameters of predicted three dimensional model of *Entodinium caudatum* cGMP-dependent protein kinase.**

| S.No | G-factor parameters | Score | Average score |
| --- | --- | --- | --- |
| 1. | Dihedral angles:  Phi-psi distribution  Chi1-chi2 distribution  Chi1 only  Chi3 & chi4  Omega | -0.31  -0.34  0.09  0.49  -0.37 | -0.21 |
| 2. | Main chain covalent forces: |  |  |
|  | Main chain bond length | 0.59 |  |
|  | Main chain bond angles | -1.32 |  |
|  |  |  | -0.52 |
|  | OVERALL AVERAGE |  | -0.27* |
| *Ideally, scores should be above -0.5. Values below -1.0 may need investigation | | | |

**Supplementary Table S7. Interactions between ligands and docked amino acid residues of *Entodinium caudatum* cGMP-dependent protein kinase.**

| S.No | Ligand | PubChem ID | Binding  energy | Ligand  efficiency | Intermole  energy | Ligand atoms (ring) | Docked amino  acid residue (bond length) |
| --- | --- | --- | --- | --- | --- | --- | --- |
| 1. | Palmitic acid | CID_985 | -4.14 | -0.23 | -8.61 | Classical Hydrogen bond:  C16-O  Alkyl hydrophobic interaction:  C2  C5-O  C8-O  C11-O  C8-O  C11-O | A chain PHE`910/HN (1.90 Å)  A chain MET`308 (4.51 Å)  A chain CYS`908 (4.54 Å)  A chain CYS`908 (4.86 Å)  A chain CYS`908 (4.52 Å)  A chain PRO`839 (4.57 Å)  A chain PRO`839 (4.26 Å) |
| 2. | Oleic acid | CID_445639 | -4.52 | -0.23 | -9.29 | Classical Hydrogen bond:  C18-OH  C18-O  C18-O  Non Classical Hydrogen bond:  C18-O  Alkyl hydrophobic interaction:  C9  C2  C6-O  C6-O  C12-O  C15-O  C12-O  Pi-Alkyl hydrophobic interaction:  C15-O  C15-O | A chain CYS`908/O (2.20 Å)  A chain ASN`909/HD22 (2.00 Å)  A chain PHE`910/HN (2.22 Å)  A chain ASN`909 (2.99 Å)  A chain LEU`308 (4.84 Å)  A chain MET`395 (4.49 Å)  A chain CYS`908 (4.95 Å)  A chain PRO`839 (4.69 Å)  A chain PRO`839 (4.30 Å)  A chain PRO`839 (5.40 Å)  A chain CYS`908 (4.53 Å)  A chain PHE`838 (5.13 Å)  A chain HIS`779 (4.63 Å) |
| 3. | Linoleic acid | CID_5280450 | -4.75 | -0.24 | -9.23 | Alkyl hydrophobic interaction:  C2-O  C9-O  C12-O  C15-O  Pi-Alkyl hydrophobic interaction:  C2  C15-O  C15-O  C15-O | A chain ARG`815 (3.88 Å)  A chain LEU`308 (5.00 Å)  A chain PRO`839 (4.19 Å)  A chain PRO`839 (4.62 Å)  A chain TYR`285 (4.93 Å)  A chain PHE`838 (5.17 Å)  A chain HIS`779 (4.51 Å)  A chain PHE`842 (4.51 Å) |
| 4. | Chlorogenate | CID_1794427 | -5.92  - | -0.24 | -9.2 | Conventional Hydrogen Bond:  C-4’OH  C-3’OH  C-3’OH  C-7OH  C-7O  C-7O  C-5O  C-4O  C-4OH  Pi-Alkyl Hydrophobic bond:  O  Miscellanous sulfur Bond:  C8 S-N | A chain SER`904/O (2.04 Å)  A chain SER`904/O (2.51 Å)  A chain PRO`903/O (2.00 Å)  A chain GLU`829/OE2 (1.81 Å)  A chain VAL`840/HN (2.65 Å)  A chain SER`837/HN (2.02 Å)  A chain SER`837/HG (2.25 Å)  A chain ARG`815/HE (2.59 Å)  A chain GLN`311/OE1 (1.84 Å)  A chain CYS`908/SG (4.92 Å)  A chain MET`395/SD (4.70 Å) |
| 5. | Dicaffeoyl quinic acid | CID_13604687 | -6.83 | -0.18 | -11.6 | Conventional Hydrogen Bond:  C-7 O  C-4 OH  C-1 OH  C-3’ OH  C-4’ OH  C-4’ O  C-3’’ OH  C-3’’ O  Pi-sigma hydrophobic interaction:  O’’  Pi-Pi stacked hydrophobic interaction:  O’’  Alkyl hydrophobic interaction:  O  Pi-Alkyl hydrophobic interaction:  O’  O’ | A chain ASN`909/HD22 (2.53 Å)  A chain ASN`909/HD21 (2.24 Å)  A chain CYS`908/SG (2.88 Å)  A chain GLU`829/OE2 (1.86 Å)  A chain GLU`829/OE2 (2.15 Å)  A chain SER`837/HN (1.91 Å)  A chain GLN`311/OE1 (2.01 Å)  A chain ARG`815/HE (1.88 Å)  A chain THR`307/CG2 (3.20 Å)  A chain TYR`285 (5.40 Å)  A chain CYS`908 (5.40 Å)  A chain PRO`903 (5.02 Å)  A chain PRO`839 (3.48 Å) |
| 6. | Quercetin-3-O-glucoside | CID_5280804 | -6.75 | -0.20 | -10.33 | Classical Hydrogen bond:  Ring A; C5-OH  C1-OH  Ring C; C4-O  C5-O  C3-OH  Ring B; C4’-OH  Ring B; C3’-OH  Pi-Alkyl hydrophobic interaction:  Ring A  Ring A  Ring C  Pi-Pi stacked hydrophobic interaction:  Ring B | A chain GLN`311/OE1 (2.02 Å)  A chain GLN`311/OE1 (1.88 Å)  A chain SER`837/HG (3.05 Å)  A chain SER`837/HG (1.63 Å)  A chain ASP`814/O (2.14 Å)  A chain HIS`779/O (2.18 Å)  A chain HIS`779/O (1.98 Å)  A chain LEU`308 (5.06 Å)  A chain PRO`839 (5.40 Å)  A chain PRO`839 (4.84 Å)  A chain PHE`838 (4.37 Å) |

**Supplementary figures**

**
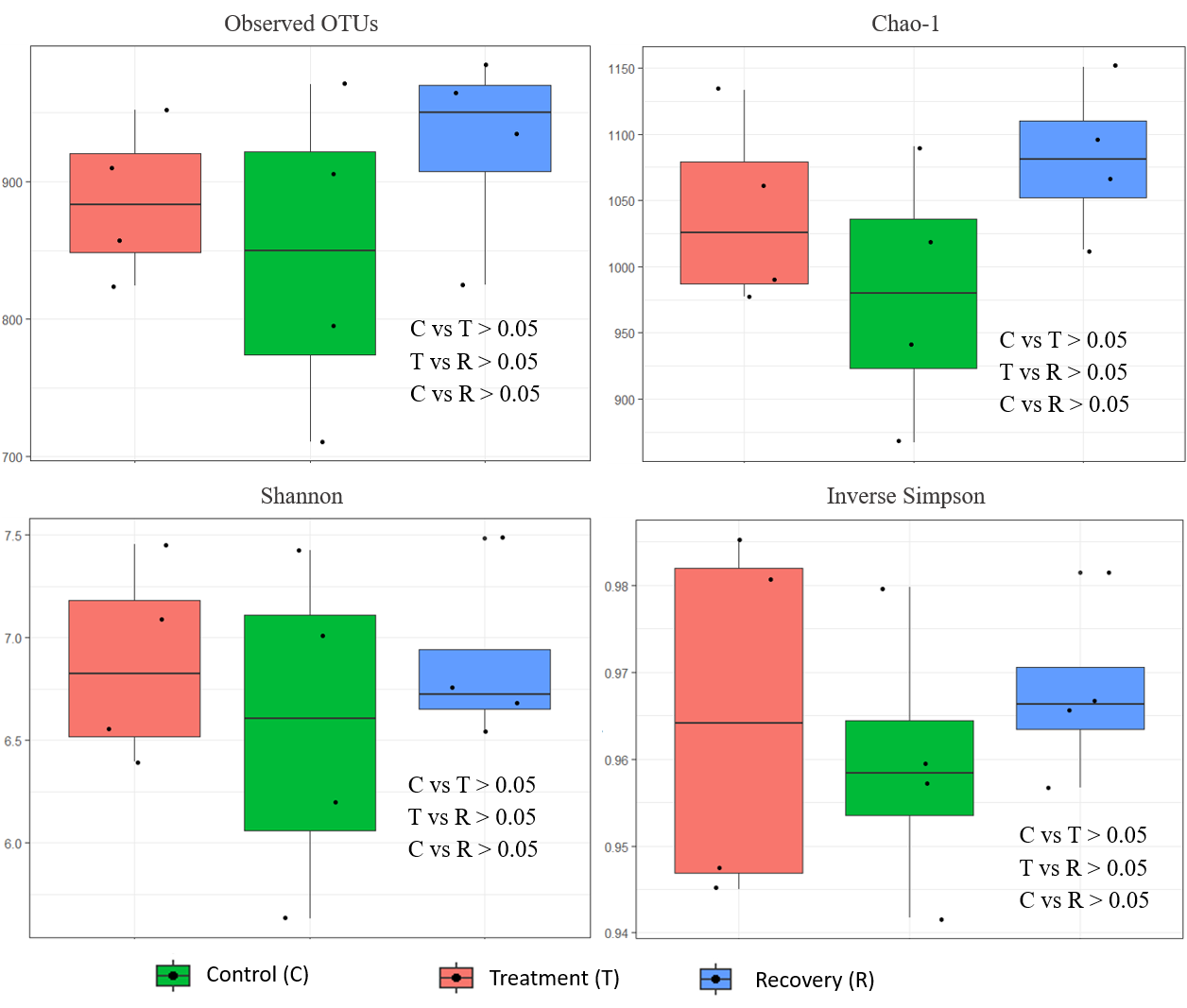
**

**Supplementary Figure S1.** Alpha diversity metrics of the bacterial/archaeal composition in the rumen of cannulated Holstein steers incubated with seeds of *P. nil in sacco*.

**
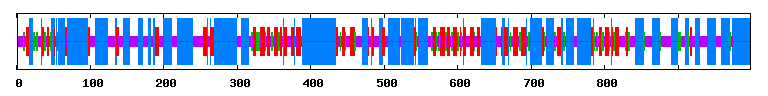
**

**Supplementary Figure S2.** Secondary structure conformation parameters of *cyclic GMP (cGMP)-dependent protein kinase* (cGK) from *E. caudatum* based on SOPMA. Helices, sheets, turns and coils are indicated with the longest, the second longest, the second shortest and the shortest vertical lines, respectively.

**
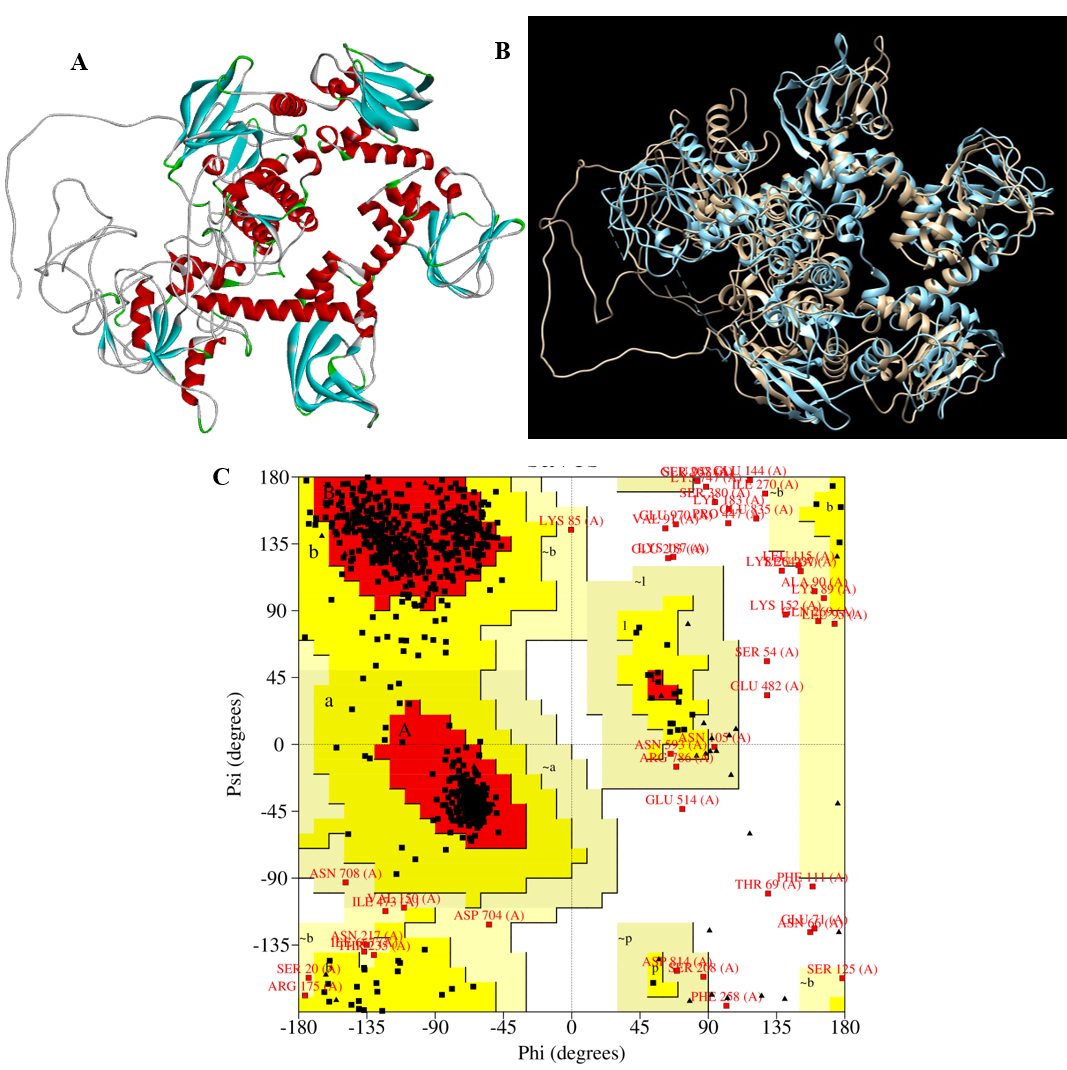
**

**Supplementary Figure S3.** (A)Three-dimensional structure of cGK. (B) Superimposition of predicted structure of cGK with the template cGK from *Plasmodium vivax* Sal-1 (PDB ID: 4RZ7). (C) Ramachandran plot of predicted cGK model.
